# PRMT1/PRMT5-Mediated Differential Arginine Methylation of CRIP1 Promotes the Recurrence of Small Cell Lung Cancer after Chemotherapy

**DOI:** 10.7150/ijbs.115225

**Published:** 2025-09-22

**Authors:** Yu Han, Lie Ma, Xiaolei Zhang, Qingyuan Ren, Qingxia Yuan, Jiawen Zhou, Yanjing Ren, Na Wan, Xin Jin, Jingyao Hou, Yanbo Wang, Baiqu Huang, Yu Zhang, Jun Lu

**Affiliations:** 1 The Key Laboratory of Molecular Epigenetics of Ministry of Education (MOE), Northeast Normal University, Changchun, 130024, China.; 2 School of life science, Northeast Normal University, Changchun, 130024, China.; 3 College of Basic Medical Science, Jilin University, Changchun, 130021, China.; 4 Department of Urology, First Hospital of Jilin University, Changchun, 130031, China.

**Keywords:** arginine methylation modification, CRIP1, inflammation, small cell lung cancer, chemotherapy, recurrence

## Abstract

Arginine methylation, a critical epigenetic modification, plays a vital role in tumor initiation and progression; however, the mechanism by which arginine methylation regulates tumor recurrence remains unclear. Here, we found the differential changes between arginine methyltransferase PRMT1 and PRMT5 in small cell lung cancer (SCLC) cells after cisplatin and etoposide treatment. PRMT5 increased at the early stage and then decreased at the later stage, while PRMT1 first decreased and then increased, which was regulated by an inflammation activated E3 ubiquitin ligase PELI1. Both PRMT5 and PRMT1 could modify the same substrate CRIP1. At the early stage, PRMT5-mediated CRIP1 R26/68 methylation activated the Wnt/β-catenin pathway to facilitate the acquisition of a stemness phenotype in senescent cells. At the later stage, PRMT1-mediated CRIP1 R16 methylation accelerated the proliferation of stem-like cells by suppressing the p38 pathway, thereby driving rapid recurrence of SCLC post-chemotherapy. Notably, combination therapy using PRMT5 inhibitor GSK3326595 along with cisplatin and etoposide significantly delayed the recurrence of SCLC. Our findings reveal the promoting effect of post-chemotherapy inflammation on tumor recurrence from an epigenetic perspective and provide a potential therapeutic strategy for SCLC treatment.

## Introduction

Cisplatin plus etoposide-based chemotherapy has been the first-line standard of care for patients with small cell lung cancer (SCLC) for several decades. Despite initial chemosensitivity, many patients experience tumor recurrence or develop therapeutic resistance 1, 2, with the median survival rarely exceeding 1 year and a 2-year survival rate of no more than 7% 3. Many antitumor agents cause therapy-induced senescence (TIS) in tumor cells, a state originally identified as an irreversible cell cycle arrest mechanism that suppresses tumorigenesis 4. However, increasing evidence suggests that cellular senescence can be reversed 5. Tumor cells undergoing TIS can escape cell cycle arrest by acquiring stem-like properties, thereby promoting cancer recurrence and metastasis. Notably, epigenetic reprogramming may serve as a critical driver of this reversal process 6, 7. Moreover, chemotherapeutic drug-induced inflammatory mediator secretion creates favorable conditions for cancer progression and recurrence 8. These inflammatory factors enhance the stemness of tumor cells, enabling them to acquire tumor-initiating capabilities 8, 9. However, the mechanisms underlying post-chemotherapy inflammation-mediated modulation of tumor cell stemness, as well as the regulatory effects of inflammation on senescence-associated stemness, remain to be further explored.

Protein arginine methylation, catalyzed by protein arginine methyltransferases (PRMTs), is a prevalent type of post-translational modification that plays a role in various physiological processes, such as cell proliferation, tumorigenesis, and tumor development 10. PRMT1 and PRMT5 are type I and type II PRMTs that catalyze asymmetric and symmetric dimethylation of arginine residues on substrates. PRMT1 and PRMT5 are expressed at high levels in many cancers including breast cancer and colorectal cancer 11, 12. PRMT1 is involved in regulating the growth, invasion, and metastasis of tumor cells in various malignancies such as breast cancer and hepatocellular carcinoma 11, 13, 14; it promotes serine synthesis and hepatocellular carcinoma growth by methylating PHGDH 14. PRMT5 plays a crucial role in tumor metastasis 11, 15, 16; it promotes tumor metastasis by methylating AKT 15. However, the role of arginine methylation in tumor recurrence remains unclear.

Cysteine-rich intestinal protein 1 (CRIP1) belongs to the LIM/double zinc finger protein family. CRIP1 is aberrantly expressed in various tumors and associated with poor prognosis 17. CRIP1 promotes invasion and migration in cervical and ovarian cancer cells by activating the Wnt/β-catenin pathway 17-19. However, studies also show that high expression of CRIP1 predicts better antitumor effects. CRIP1 expression is correlated with favorable prognosis and decreased metastasis in patients with osteosarcoma 20, and CRIP1 inhibits cell proliferation in breast cancer 21. However, the role of CRIP1 in the development and progression of SCLC, as well as its function in response to chemotherapy, has not been reported.

In this study, we found that recurrence of SCLC after chemotherapy is accompanied by dynamic changes in inflammation and arginine methylation. We also showed that PRMT1 and PRMT5 mediated differential CRIP1 arginine methylation, which promoted SCLC recurrence after chemotherapy. Post-chemotherapy inflammation modulated the differential expression of PRMT1 and PRMT5 by activating the E3 ubiquitin ligase PELI1. Cisplatin and etoposide combined with the PRMT5 inhibitor GSK3326595 effectively delayed the recurrence of SCLC, suggesting its potential as a promising new drug candidate for delaying SCLC recurrence in patients undergoing chemotherapy.

## Materials and Methods

### Cell lines and reagents

All cell lines were obtained from the American Type Culture Collection. NCI-H446, NCI-H82 and NCI-H1688 cells were cultured in RPMI-1640 medium supplemented with 10% fetal bovine serum (FBS) (Procell, China). DMS114 and HEK-293T cells were cultured in Dulbecco's modified Eagle's medium (Sigma-Aldrich) with 10% FBS. All the cell lines were cultured at 37 °C in 5% CO_2_. Stem-like cells were cultured in DMEM/F12 supplemented with 20 ng/ml EGF (Sigma-Aldrich, E9644), 1×B27 (Gibco, 17504044), 20ng/ml bFGF (Peprotech, 100-18B), and 10ng/ml insulin (MCE, HY-P73243). Anakinra (HY-108841), GSK3326595 (HY-101563), Adezmapimod (HY-10256), XAV-939 (HY-15147), MG132 (HY-13259) and C-7280948 (HY-15890) were purchased from MCE. CHX (C104450), Cisplatin (232120) and Etoposide (E1383) were purchased from Sigma-Aldrich.

### Antibodies and plasmids

The description of antibodies was provided in Table S1.

shPRMT1 and Flag-PRMT1 expression plasmids were constructed, as described previously 22. The following plasmids were utilized in the study: pCDH-CMV-3×Flag-PRMT5, pCDH-CMV-3×Flag-CRIP1, pCDH-CMV-3×Flag-PELI1, pCDH-CMV-3×Flag-PELI2, pCDH-CMV-3×Flag-PELI3, pCDH-CMV-3×Flag-TRAF6, pCDH-CMV-3×Flag-SMURF2, pCDH-CMV-3×Flag-VHL, pCDH-CMV-3×Flag-BTRC, pCDH-CMV-HA-p38 and pCDH-CMV-HA-PRMT5. Flag-tagged CRIP1 plasmids was used to generate Flag-tagged CRIP1 R16K, R26K, R68K, R26/68K, R16F and R26/68F plasmids. The bacterial expression plasmid, which expresses glutathione S-transferase (GST)-labeled CRIP1, its mutants GST-CRIP1 R16K and GST-CRIP1 R26/68K, as well as the truncated forms GST-CRIP1 (1-57aa) and GST-CRIP1 (58-77aa), was prepared by inserting the target DNA fragments into the pGEX-6P-1 vector.

Additionally, interference sequences targeting PRMT5, PELI1, PELI2, PELI3, TRAF6, VHL, CRIP1, FLNB, SVIL and MyD88 were designed and cloned into the lentiviral RNAi system pLKO.1. The shRNA sequences are listed in Table S2.

### RNA-seq analysis

RNA-seq analysis was conducted as described 22. Samples (n=3 per group) were collected from the 0-day pre-chemotherapy group and post-chemotherapy groups at drug withdrawal on days 5, 8, 17, and 30 (W5, W8, W17, W30) for RNA-seq analysis. mRNA library construction and sequencing were performed by the BGI Institute of Life Sciences (Wuhan, China).

### Co-immunoprecipitation and western blot analyses

Cells were collected and lysed in Buffer A [25 mM Tris-HCl (pH 8.0), 10 mM NaCl, 1 mM EDTA, and 0.5% NP-40] for 30 minutes. The supernatant obtained after centrifugation was incubated overnight (4 °C, vertical mixer) with 2-5 μg of antibodies, followed by addition of 30-40 μl of Pure Proteome Protein A/G Mix Magnetic Beads (Millipore, LSKMAGAG02) at room temperature for 2-4 h. The magnetic beads were washed with pre-chilled phosphate-buffered saline (PBS), resuspended in 60-80 μl of loading buffer, and subjected to western blotting analysis as described 22.

### *In vitro* methylation assay

*In vitro* methylation assay was performed as previously described 23.

### RNA extraction, reverse transcription and real-time RT-qPCR analysis

Detailed protocols have been reported previously by our group 22. All primer sequences used for RT-qPCR analysis are listed in Table S3.

### Immunofluorescence

Immunofluorescence was performed as previously described 22.

### SA-β-gal staining

SA-β-gal staining was performed as previously described 24.

### Colony formation assay

NCI-H446 cells were suspended and seeded into each well of a 6-well plate for culture. The culture medium collected on day W8 after chemotherapy was used to continuously treat the cells from day W23 to day W30. On day W30, the colonies were fixed, stained with crystal violet, and the colony formation rate was calculated.

### Sphere-formation analysis

10,000 cells were seeded into an ultra-low attachment plate and cultured using a specialized stem cell medium. The size of the tumor spheroids was observed under a light microscope and the count of spheres with a diameter greater than 50 μm was counted.

### Flow cytometric analysis

A total of 1×10⁶ cells per sample were harvested and subsequently washed with PBS. For stemness marker detection, the cell surface was stained using CD44 (BD Pharmingen, 559942) antibody. For senescence detection, intracellular staining was performed with p21 antibody (Proteintech, 10355-1-AP). The validity of these antibodies for their intended applications was confirmed by the manufacturers. For the detection of Side Population (SP) cells, Hoechst 33342 dye (MCE, HY-15559) was added to achieve a final concentration of 5 μg/ml, and the cells were co-incubated with the dye at 37℃ for 90 minutes. The control group cells were first pre-incubated with 100 µM verapamil (MCE, HY-14275) for 15 minutes before adding Hoechst 33342 dye. SP cells were visualized by red vs. blue ultraviolet channels. All analyses were conducted using flow cytometry with a Beckman Coulter CytoFLEX flow cytometer, and the proportion of positive cells was quantified using FlowJo™ X software.

### Soft agar colony formation assay

After treating NCI-H446 cells with C+E (Cisplatin plus Etoposide), a 14-day stem cell sphere formation process was initiated on day W8. The cells with stem-like characteristics were enriched, digested into a single-cell suspension, and then mixed with 0.7% agarose, which was layered over a bottom layer of 1.2% agarose. Visible colonies were scored 14 days later using an Olympus SZX12 stereomicroscope.

### Protein purification and GST pull-down assay

The target gene with a Flag tag was transiently transfected into HEK-293T cells. After 48 hours, the cells were lysed, and Flag affinity gel (Selleck, B23102) was added for incubation at 4 ℃ for 4 hours. Subsequently, the Flag-tagged protein was purified through competitive elution using 3× FLAG peptide (MCE, HY-P0319). GST pull-down assay was performed as described 23. GST-tagged CRIP1 (wild-type, mutants, truncations) and GST-p38 were expressed in BL21 and induced with 1 mM IPTG at 4 °C for 16 h.

### Mouse xenograft model

For xenograft establishment, a suspension containing 2×10⁶ NCI-H446 cells was subcutaneously injected into the right upper axillary region of nude mice. The longest (a) and perpendicular shortest (b) diameters of the tumors were measured using a vernier caliper, and the tumor volume was calculated using the formula: Tumor Volume (V)=ab²/2. Mice were randomized into groups once tumor volumes reached 100-200 mm³. Each treatment cycle lasted 7 days. On day 1, cisplatin was administered intraperitoneally at a dose of 5 mg/kg; on days 1, 2, and 3, etoposide was administered intraperitoneally at a dose of 10 mg/kg; and on days 1, 2, and 3, GSK3326595 was administered orally via gavage at a dose of 40 mg/kg. The treatment regimen was continued for two cycles. Prior to chemotherapy, 500 μl of sodium lactate Ringer's solution was subcutaneously injected into the mice to alleviate renal toxicity induced by the chemotherapeutic agents. These animal experiments were approved by the Animal Care Committee of Northeast Normal University, China.

### Histological analyses

After fixation in formalin, the tissue samples underwent paraffin embedding and tissue sectioning. Subsequently, the tissues were deparaffinized and rehydrated. HE staining was carried out according to the instructions of the HE staining kit (Absin, catalog no. abs9217). Immunohistochemistry (IHC) staining was performed as previously described 24.

### Statistical analysis

The results were compiled from at last three independent replicate experiments and are presented as mean ± SD. Differences between groups were assessed by an unpaired Student's t test for two groups or one-way analysis of variance (ANOVA) followed by Tukey`s post hoc test for multiple groups. Statistical analysis was performed using GraphPad Prism software.

## Results

### Recurrence of SCLC is accompanied by dynamic inflammatory changes after chemotherapy

**Figure 1:**
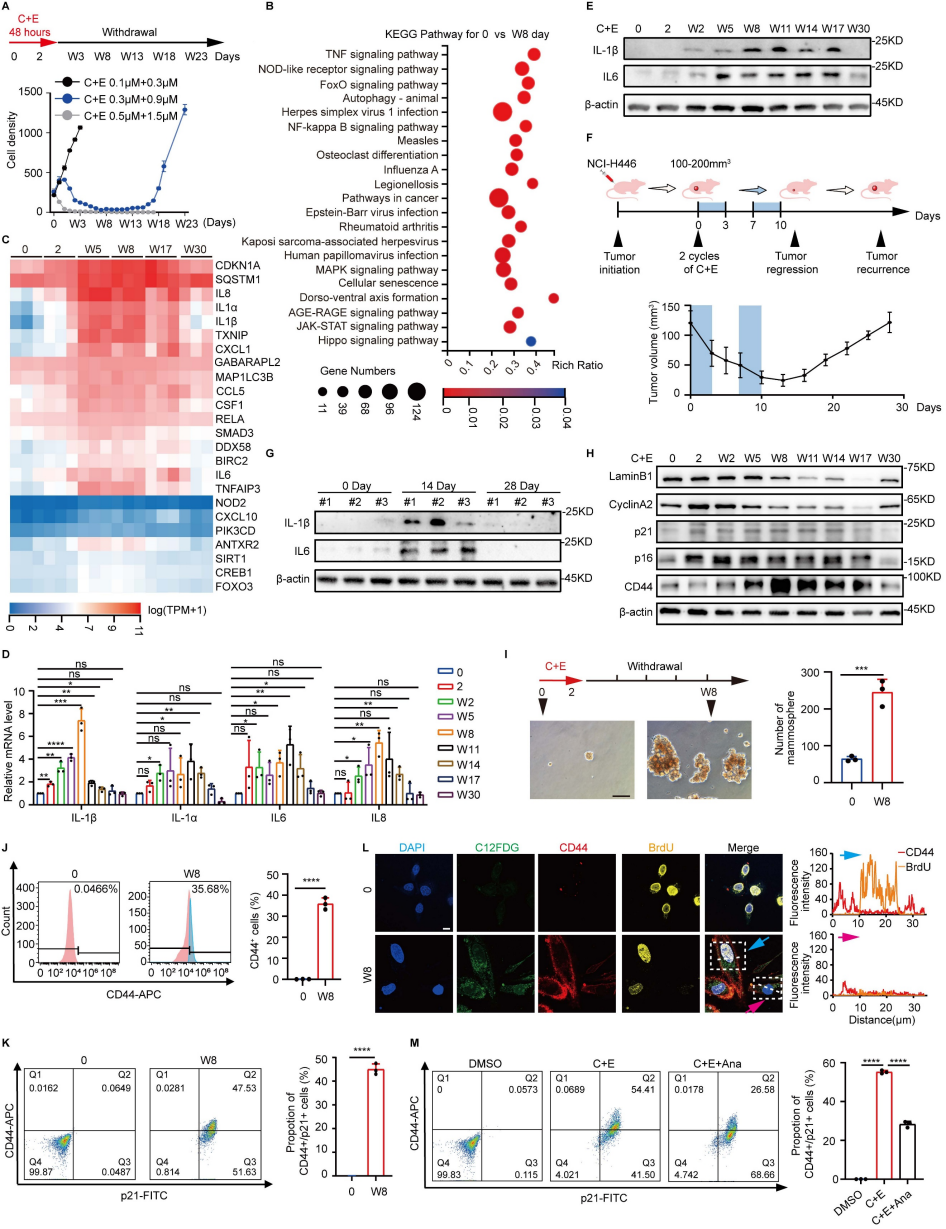
** Recurrence of SCLC is accompanied by dynamic inflammatory changes after chemotherapy.** (A) NCI-H446 cell survival curves: Cells were treated with cisplatin plus etoposide (C+E) for 48 h, followed by drug withdrawal (W). X-axis: time points; Y-axis: cell count per 50× field. (B) Differential enrichment of Kyoto Encyclopedia of Genes and Genomes signaling pathways in NCI-H446 cells on day W8 post-chemotherapy compared to before chemotherapy. (C) Heatmap analysis of mRNA level changes in genes of NCI-H446 cells at different time points after chemotherapy treatment. (D) RT-qPCR assessment of IL-1β, IL-1α, IL6, and IL8 mRNA levels in NCI-H446 cells before and after chemotherapy. (E) Western blot detection of IL-1β and IL6 expression in NCI-H446 cells at multiple time points pre/post-chemotherapy. (F) NCI-H446 xenograft model: Changes in tumor volume pre/post-chemotherapy were monitored (n=5 mice). (G) Western blot analysis of IL-1β and IL6 proteins in NCI-H446 xenograft tumors collected pre-treatment, on day 14, and on day 28 post-treatment. (H) Western blot analysis of senescence and stemness markers in NCI-H446 cells at multiple time points pre/post-chemotherapy. (I) Sphere-forming ability of NCI - H446 cells before chemotherapy and NCI-H446 cells on day W8 post-chemotherapy (scale bar: 100 μm). (J) Flow cytometry detection of CD44-positive NCI-H446 cells before chemotherapy and on day W8 after chemotherapy. (K) Flow cytometry detection of changes in the proportion of CD44⁺/p21⁺ double-positive NCI-H446 cells before chemotherapy and on day W8 after chemotherapy. (L) BrdU incorporation in NCI-H446 cells before chemotherapy and on day W8 after chemotherapy: analysis of BrdU and CD44 fluorescence in C12FDG-positive cells. Blue arrows: BrdU-positive senescent cells; pink arrows: BrdU-negative senescent cells (Scale bars: 10 μm). (M) The proportion of CD44⁺/p21⁺ double-positive cells in NCI-H446 cells treated with a combination of C+E along with Ana on day W8 after chemotherapy was analyzed using flow cytometry. Data are shown as means ± SD. ns, not significant; *P < 0.05; **P < 0.01; ***P < 0.001; ****P < 0.0001.

To explore the mechanisms underlying the recurrence of SCLC after chemotherapy, we first simulated the process of SCLC recurrence after chemotherapy at the cellular level. NCI-H446 cells were treated with different doses of cisplatin and etoposide (C+E) for 48 hours, followed by drug withdrawal (W). The results showed that the optimal combination, mimicking clinical SCLC recurrence, was 0.3 μM cisplatin and 0.9 μM etoposide, which induced a transition from proliferation inhibition to re-proliferation (Fig. 1A). The mechanisms of SCLC recurrence were examined by performing transcriptome analysis at various stages after chemotherapy. Analysis of Kyoto Encyclopedia of Genes and Genomes pathways showed enrichment of inflammatory-associated pathways, specifically nuclear factor-κB and tumor necrosis factor on day W8 compared to baseline (Fig. 1B). A heatmap further revealed dynamic changes in inflammatory factors associated with recurrence after chemotherapy. The levels of factors such as interleukin (IL)-1β, IL-1α, IL6, and IL8 increased initially on day W5, followed by a gradual decrease after W17 (Fig. 1C). This dynamic change occurred at both the protein and mRNA levels (Fig. 1D and E, Fig. S1A-D). We generated an NCI-H446 xenograft mouse model to confirm these changes* in vivo*, and the results also showed altered levels of IL-6 and IL-1β (Fig. 1F and G), confirming the changes in inflammatory factors that occur during SCLC recurrence after chemotherapy.

In addition to inflammation-related pathways, cell senescence and the Hippo signaling pathway, which is related to stemness, were significantly enriched following chemotherapy (Fig. 1B). Detection of senescence markers showed a decrease in cell proliferation associated with senescence in SCLC, which was followed by re-proliferation after chemotherapy (Fig. 1H, Fig. S1E-K). Detection of stemness-related indicators showed that the stemness phenotype of SCLC was significantly enhanced after chemotherapy (Fig. 1H-J, Fig. S1E-G and L-N). Furthermore, analysis of the stemness characteristics of senescent SCLC cells after chemotherapy showed a significant increase in CD44 fluorescence intensity associated with cellular senescence, and more than 40% of the senescent cells acquired stem-like characteristics. (Fig. 1K, Fig. S1O). BrdU incorporation demonstrated that these senescent cells with acquired stemness phenotypes possessed potential proliferative capacity (Fig. 1L). These findings indicate that the acquisition of senescence-associated stemness may promote SCLC recurrence after chemotherapy. Notably, inhibition of the post-chemotherapy inflammatory response using Anakinra (Ana), a receptor antagonist for IL1 25, significantly suppressed the ability of senescent cells to acquire stemness (Fig. 1M, Fig. S1P-R). The results suggest that the elevation of inflammation following chemotherapy promotes the acquisition of a stemness phenotype by senescent SCLC cells, which could be a crucial factor driving SCLC recurrence after chemotherapy.

### Inflammation levels regulate the differential alterations of PRMT1 and PRMT5

**Figure 2:**
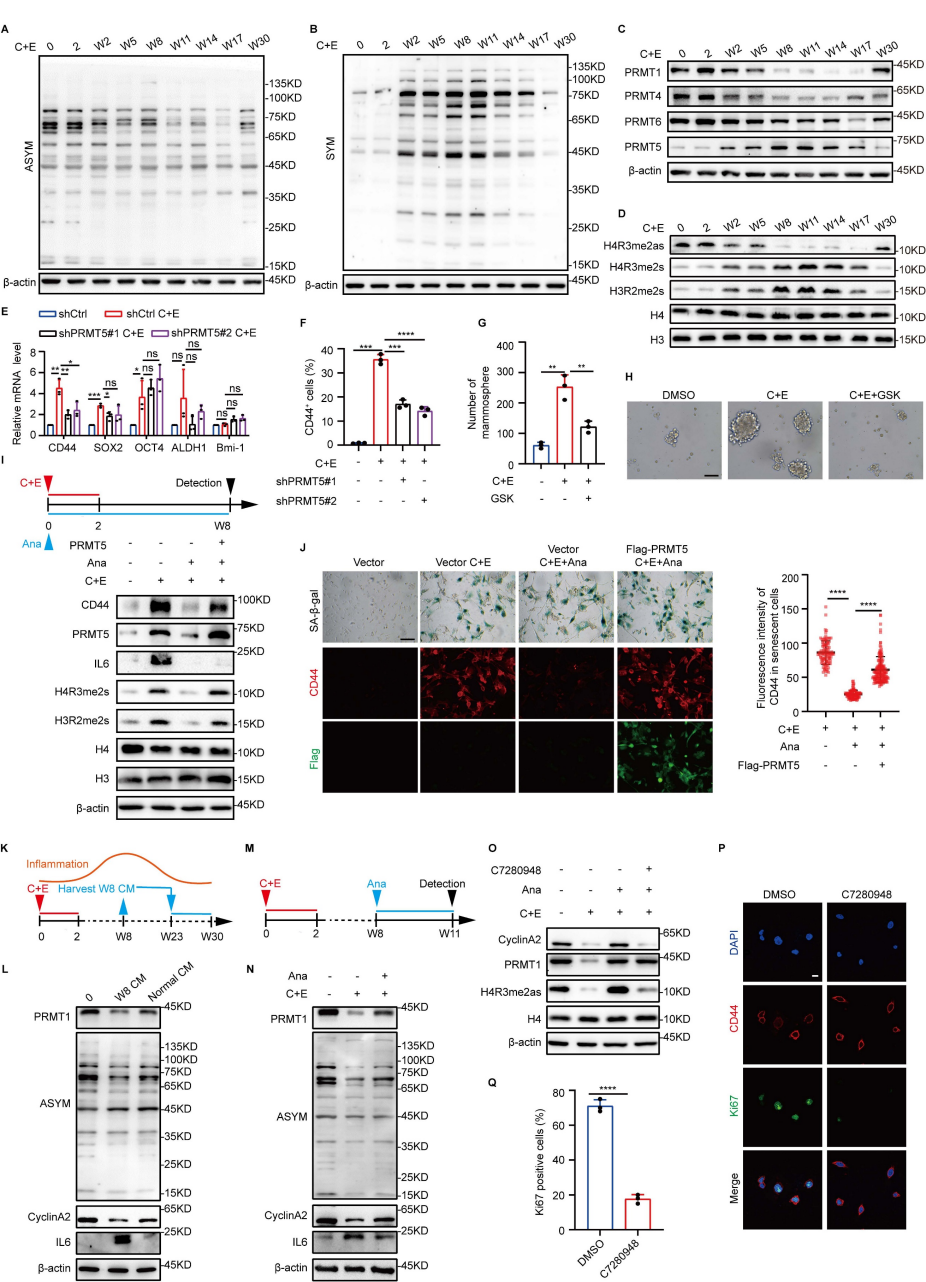
** Inflammation levels regulate the differential alterations of PRMT1 and PRMT5.** (A and B) Western blot analysis of ASYM and SYM levels in NCI-H446 cells at indicated time points after chemotherapy. (C and D) Western blot analysis of arginine methyltransferases and their specific modified substrates, including H4R3me2as, H4R3me2s and H3R2me2s in NCI-H446 at corresponding time points after chemotherapy. (E) The mRNA levels of stemness genes in NCI-H446 were detected on day W8 after PRMT5 knockdown and chemotherapy treatment. (F) Flow cytometry was used to quantify CD44-positive cells in NCI-H446 cells post-PRMT5 knockdown on day W8 after chemotherapy. (G and H) On day W8, NCI-H446 cells treated with GSK3326595 (GSK) and C+E were subjected to a stem cell sphere formation assay (scale bar: 100 μm). (I) After overexpression of PRMT5 in NCI-H446 cells followed by treatment with C+E+Ana, the expression levels of PRMT5, CD44, H4R3me2s, and H3R2me2s were detected on day W8 by Western blot. (J) NCI-H446 cells overexpressing PRMT5 were treated with C+E+Ana, with Ana being added from 0 to W8, and CD44 fluorescence intensity in SA-β-gal-positive cells was quantified on day W8 (scale bar: 100 μm). (K and L) Western blot analysis of PRMT1, ASYM, CyclinA2 and IL6 was performed on day W30 in NCI-H446 cells that were continuously treated with medium collected from W8 cells during the period from W23 to W30. (M and N) Western blot analysis of PRMT1, ASYM, CyclinA2 and IL6 expression in NCI-H446 cells treated with Ana from W8 to W11. (O) Western blot analysis of PRMT1, H4R3me2as and CyclinA2 on day W11 in NCI-H446 cells co-treated with Ana and C7280948 from W8 to W11. (P and Q) Stem cell sphere formation was conducted in cells at W8 post-chemotherapy for 14 days. The enriched stem-like cells were then treated with C7280948 for 72 hours, followed by statistical analysis of Ki67-positive proportions in the stem-like cells (scale bar: 10 μm). Data are shown as the mean ± SD. *P < 0.05, **P < 0.01, ***P < 0.001 and ****P < 0.0001.

To explore the mechanisms by which inflammation influences senescence-associated stemness after chemotherapy, we screened various types of epigenetic modifying enzymes. Arginine asymmetric dimethylation (ASYM) and symmetric dimethylation (SYM) exhibited differential dynamic changes after drug administration (Fig. 2A and B, Fig. S2A-D). Examination of arginine methyltransferases showed that after chemotherapy, the expression trend of PRMT1 correlated with that of ASYM, with an initial decrease followed by a gradual increase, whereas the expression trend of PRMT5 correlated with that of SYM, exhibiting the opposite pattern (Fig. 2C, Fig. S2E-G). Moreover, the expression patterns of substrates specifically modified by PRMT1 (H4R3me2as) and PRMT5 (H4R3me2s and H3R2me2s) further highlighted the dynamic differences in the activities of PRMT1 and PRMT5 post-chemotherapy (Fig. 2D). These results suggest that PRMT1 and PRMT5 may function at distinct stages during SCLC recurrence after chemotherapy.

After chemotherapy, the rise in both PRMT5 expression and activity, which coincided with enhanced SCLC stemness, prompted us to further study PRMT5's impact on SCLC stemness post-chemotherapy. When PRMT5 was knocked down or combined with the PRMT5 inhibitor GSK3326595 (GSK), the stemness characteristics of SCLC after chemotherapy were significantly inhibited (Fig. 2E-H, Fig. S2H-N). These results indicate that PRMT5 promotes the stemness characteristics of SCLC after chemotherapy. Cells were then treated with Ana to analyze the correlation between inflammation and PRMT5 after chemotherapy. The results showed a significant decrease in PRMT5 expression and activity in response to the decrease in inflammatory levels after chemotherapy (Fig. 2I). Notably, overexpression of PRMT5 can reverse the reduction in stemness of senescent cells caused by inflammation inhibition (Fig. 2I and J). Taken together, these results suggest that increased inflammation at the early stage promotes the acquisition of stem-like phenotypes in senescent cells by upregulating PRMT5 expression.

In our model, inflammation exhibits dynamic changes, and existing studies have reported that inflammation negatively regulates the proliferation of neural stem cells 26. Next, we investigated the impact of reduced inflammation levels during the later stages of recurrence on the proliferation of SCLC stem-like cells. We first used a W8 high-level inflammatory medium to increase inflammation levels in the late recurrence stage, which showed inhibition of the recurrence process, along with decreased expression of cyclinA2 and PRMT1 (Fig. 2K and L, Fig. S2O). Conversely, treatment of W8 cells with Ana to rapidly reduce inflammation levels to the low-inflammatory state observed during recurrence upregulated the expression of PRMT1 and cyclinA2 (Fig. 2M and N). However, when a PRMT1 inhibitor, C7280948, was further added along with Ana, CyclinA2 expression was significantly inhibited (Fig. 2O), which indicated that the elevation of PRMT1 expression caused by the decrease in inflammation levels may be the key factor promoting the proliferation of SCLC cells in the late recurrence stage. We then assessed the impact of PRMT1 on the proliferation of stem-like cells in the late recurrence stage. Both PRMT1 knockdown and activity inhibition significantly decreased the proportion of Ki67-positive stem-like cells and the number of cell clones (Fig. 2P and Q; Fig. S2P-S). These results suggest that the decrease in inflammation during the late recurrence stage further promotes the proliferation of stem-like cells by upregulating PRMT1 expression.

### PRMT1 competes with PRMT5 for the methylation of the substrate CRIP1

**Figure 3:**
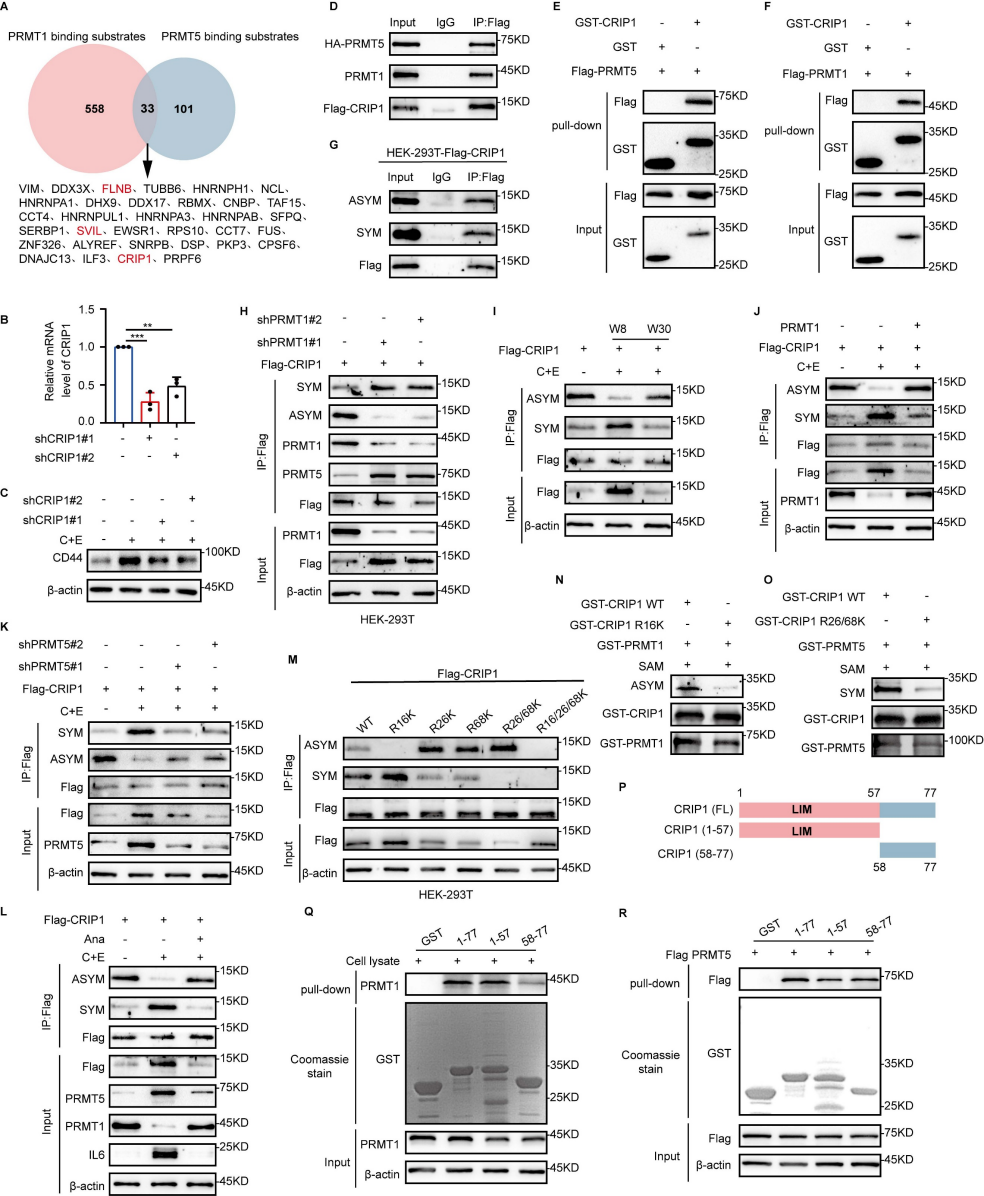
** PRMT1 competes with PRMT5 for the methylation of the substrate CRIP1.** (A) Analysis of the mass spectrometry data to identify common substrates of PRMT1 and PRMT5. (B) RT-qPCR analysis of CRIP1 interference efficiency in NCI-H446. (C) Western blot analysis of CD44 protein expression in CRIP1-knockdown cells on day W8 after chemotherapy. (D) Co-immunoprecipitation and immunoblot analysis of interaction among CRIP1, PRMT1 and PRMT5 in HEK-293T cells transfected with Flag-CRIP1 and HA-PRMT5. (E and F) GST-pulldown assay for binding of CRIP1 to PRMT5 and PRMT1. (G) Western blot analysis of ASYM and SYM modifications of CRIP1 in Flag-CRIP1-transfected HEK-293T cells. (H) PRMT1 was knocked down in Flag-CRIP1-transfected HEK-293T cells, followed by co-immunoprecipitation and immunoblot analysis of CRIP1 arginine methylation modification levels and its binding with PRMT1 or PRMT5. (I) Western blot analysis of CRIP1 arginine methylation modification levels at indicated time points after chemotherapy in NCI-H446. (J and K) Co-immunoprecipitation and immunoblot analysis of ASYM and SYM modifications of CRIP1 in NCI-H446-Flag-CRIP1 cells overexpressing PRMT1 or knocked down PRMT5 on day W8. (L) NCI-H446 cells were treated with C+E+Ana, and Western blot analysis of arginine methylation modification of CRIP1 on day W8. (M) Co-IP analysis of CRIP1 arginine methylation levels in HEK-293T cells transfected with Flag-CRIP1 WT and corresponding arginine site mutants. (N and O) Western blot analysis of CRIP1 methylation modification after incubating GST-PRMT1/5 with GST-CRIP1 WT or mutants in the presence of SAM. (P to R) Schematic diagram shows the full-length (FL) and truncated mutant schematic structures of CRIP1. GST-pulldown assays with GST-CRIP1 and truncation mutants incubation with HEK-293T lysate or PRMT5-overexpressing HEK-293T lysate. Analysis of PRMT1 and PRMT5 interaction with CRIP1 domains. Data are shown as the mean ± SD. **P < 0.01 and ***P < 0.001.

PRMT1 and PRMT5 may synergistically promote tumorigenesis by inducing distinct modifications on the same substrate 27. To screen for common substrates of PRMT1 and PRMT5, we referred to the mass spectrometry data on their binding from published articles (Fig. 3A) 28-32. Among the substrates identified, FLNB, SVIL, and CRIP1 are associated with stem-like cell characteristics 17, 33, 34. Among these proteins, CRIP1 knockdown significantly downregulated CD44 expression (Fig. 3B and C, Fig. S3A-D), suggesting that CRIP1 may play a crucial role in the acquisition of stemness in SCLC.

We found that PRMT1 and PRMT5 bind directly to CRIP1 (Fig. 3D-F, Fig. S3E and F), and CRIP1 underwent both SYM and ASYM of arginine residues (Fig. 3G). Knockdown of PRMT1 decreased the ASYM modification of CRIP1 and increased the SYM modification, promoting the interaction between CRIP1 and PRMT5 (Fig. 3H). After knockdown of PRMT5, the SYM modification of CRIP1 was decreased while the ASYM modification was increased, and the binding of CRIP1 to PRMT1 was enhanced Fig. S3G). These results indicate that PRMT1 and PRMT5 may competitively modify the substrate CRIP1. Furthermore, the methylation modifications ASYM and SYM in CRIP1 exhibited differential dynamic changes in the recurrence model (Fig. 3I). Detection of arginine methylation modifications of CRIP1 after chemotherapy, with overexpression of PRMT1 or interference of PRMT5, further demonstrated the regulatory effects of PRMT1 and PRMT5 on CRIP1 methylation modifications (Fig. 3J and K), and methylation of CRIP1 was highly responsive to inflammatory changes after chemotherapy (Fig. 3L).

To identify the methylation sites of CRIP1 modified by PRMT1 and PRMT5, we focused on three arginine residues that are highly conserved across species (Fig. S3H). Mutation of R16 to lysine (K) significantly reduced ASYM, whereas single or double mutations of R26 and R68 to K markedly decreased SYM levels. The decrease in ASYM (or SYM) was accompanied by an increase in SYM (or ASYM), confirming the competitive modification of CRIP1 by PRMT1 and PRMT5 (Fig. 3M). *In vitro* methylation assays showed that the R16K mutation significantly inhibited CRIP1 ASYM, whereas the R26/68K mutations significantly inhibited CRIP1 SYM (Fig. 3N and O). These results suggest that R16 is a site for PRMT1-mediated arginine methylation, while R26/68 are sites for PRMT5-mediated arginine methylation. Structural domain analysis of CRIP1 showed that PRMT1 binds strongly to the LIM domain (aa1-57), whereas PRMT5 binds to both the LIM domain (aa1-57) and the C-terminal domain (aa58-77) (Fig. 3P-R). The binding regions align with the methylation sites, further supporting our findings. The research findings consistently indicate that PRMT1 and PRMT5 catalyze arginine methylation on R16 and R26/68 sites of CRIP1, respectively.

### PRMT5-mediated symmetric dimethylation of CRIP1 R26/68 promotes the acquisition of stemness phenotype

**Figure 4:**
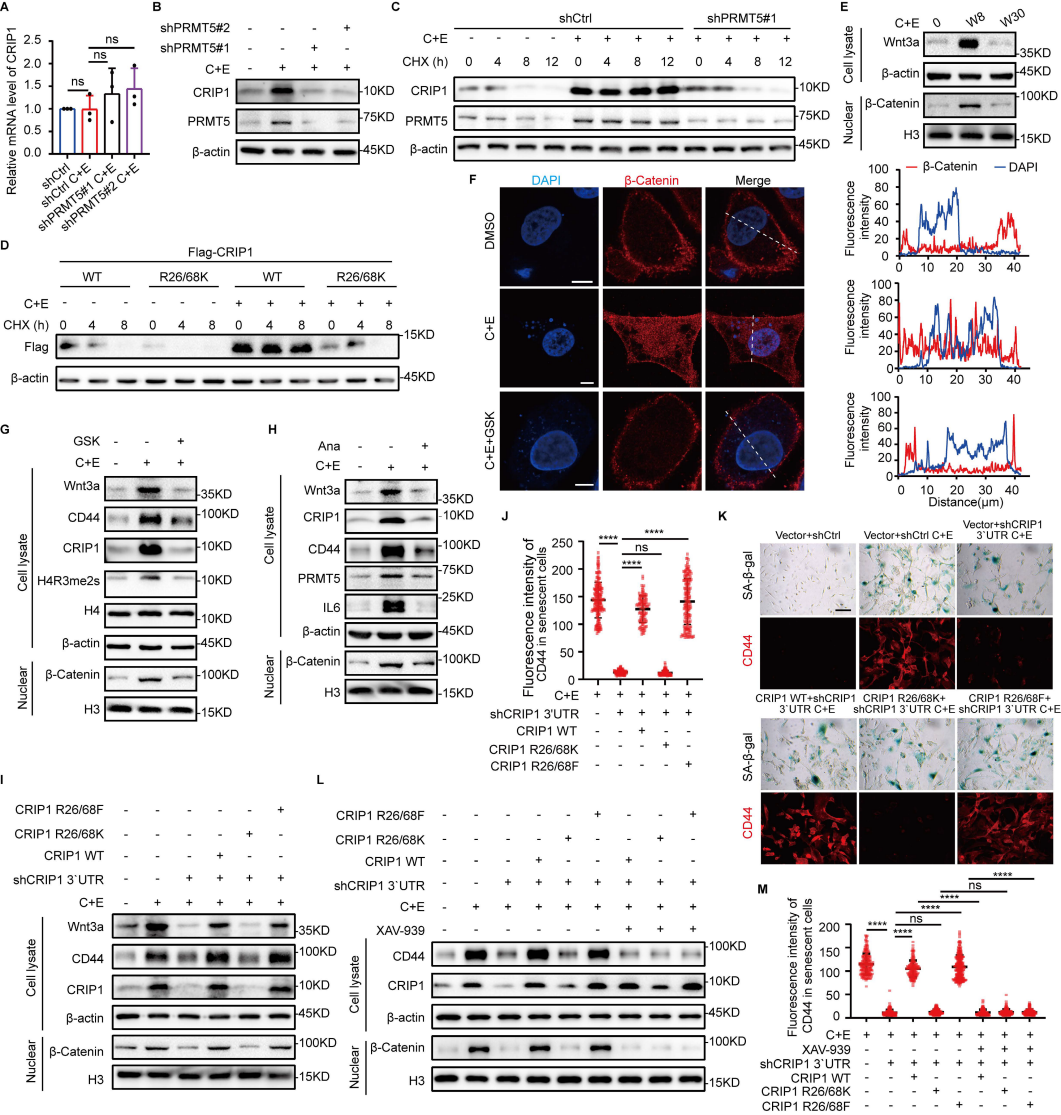
** PRMT5-mediated symmetric dimethylation of CRIP1 promotes the acquisition of stemness phenotype.** (A) In NCI-H446 cells with PRMT5 knocked down, RT-qPCR analysis of CRIP1 in W8 cells after chemotherapy. (B) Western blot analysis of CRIP1 protein expression on day W8 in NCI-H446 cells with PRMT5 knocked down. (C) CHX was added to W8 cells with PRMT5 knocked down, and Western blot analysis of CRIP1 half-life in NCI-H446 cells. (D) CHX-treated W8 cells overexpressing Flag-CRIP1 WT or R26/68K were analyzed by Western blot for Flag expression in NCI-H446 cells. (E) Western blot analysis of Wnt3a and nuclear β-Catenin in NCI-H446 cells before and after chemotherapy. (F) Immunofluorescence detected β-Catenin localization in NCI-H446 cells treated with C+E+GSK on day W8 (scale bar: 10 μm). (G and H) Western blot analysis of Wnt3a, CD44, and CRIP1 in total lysate, and nuclear β-Catenin on day W8 in NCI-H446 cells treated with C+E+GSK or C+E+Ana. (I) Western blot analysis of Wnt3a, CD44, CRIP1 in total lysate, and nuclear β-Catenin on day W8 in NCI-H446 cells with CRIP1 knocked down and overexpressing CRIP1 WT, R26/68K, or R26/68F. (J and K) CD44 fluorescence was analyzed in SA-β-gal-positive NCI-H446 cells on day W8 using SA-β-gal and CD44 co-staining (scale bar: 100 μm). (L) Western blot analysis of CD44 and CRIP1 in total lysate, and nuclear β-Catenin on day W8 in NCI-H446 cells treated with C+E+XAV-939. (M) CD44 fluorescence was analyzed in SA-β-gal-positive NCI-H446 cells treated with C+E+XAV-939 on day W8 using SA-β-gal and CD44 co-staining (scale bar: 100 μm). Data are shown as the mean ± SD. ****P < 0.0001.

Our studies show that interference with PRMT5 decreases the SYM modification of CRIP1 and significantly downregulates CRIP1 expression (Fig. S3G). To further explore the regulation of CRIP1 by PRMT5, we knocked down PRMT5, which downregulated CRIP1 protein expression without affecting CRIP1 mRNA levels (Fig. 4A and B). In addition, silencing PRMT5 shortened the half-life of CRIP1 after chemotherapy (Fig. 4C). Strikingly, mutation of the R26/68 sites methylated by PRMT5 on CRIP1 led to a shortened half-life of CRIP1 (Fig. 4D). These results suggest that PRMT5-mediated R26/68 methylation stabilizes CRIP1 protein level.

The activation of the Wnt/β-catenin pathway is a key driver in conferring a stemness phenotype on senescent cells (6, 7, 35. Therefore, we further explored the regulatory effect of PRMT5-mediated methylation modification at the R26/68 sites of CRIP1 protein on the Wnt/β-catenin pathway. We examined the expression of components of the Wnt/β-catenin pathway during the recurrence process and found a significant increase in Wnt3a expression and nuclear translocation of β-catenin in W8 cells (Fig. 4E, Fig. S4A). Treatment with the GSK or Ana markedly inhibited Wnt3a expression and the nuclear translocation of β-catenin (Fig. 4F-H). These results suggest that PRMT5 is involved in the activation of the Wnt/β-catenin pathway. To investigate the activation of the Wnt/β-catenin pathway by methylation at the CRIP1 R26/68 sites, we overexpressed wild-type CRIP1 (WT), mutant CRIP1 R26/68K, and CRIP1 with simulated methylation (R26/68F), and then measured the activity of the Wnt/β-catenin pathway. Both CRIP1 WT and the R26/68F activated the Wnt/β-catenin pathway, promoting the nuclear translocation of β-catenin and enhancing the stemness phenotype of senescent cells. However, the R26/68K mutant had no effect on the activation of the stemness pathway or related stemness characteristics (Fig. 4I-K, Fig. S4B-E). Inhibition of the Wnt/β-catenin pathway by VAX-939 suppressed the effect of CRIP1 WT and R26/68F on promoting the expression of stemness genes and the acquisition of stemness in senescent cells (Fig. 4L and M, Fig. S4F). These results suggest that PRMT5-mediated R26/68 symmetric methylation of CRIP1 promotes the acquisition of a stemness phenotype in senescent cells through the activation of the Wnt/β-catenin pathway.

### PRMT1-mediated asymmetric dimethylation of CRIP1 R16 promotes proliferation of stem-like cells

**Figure 5:**
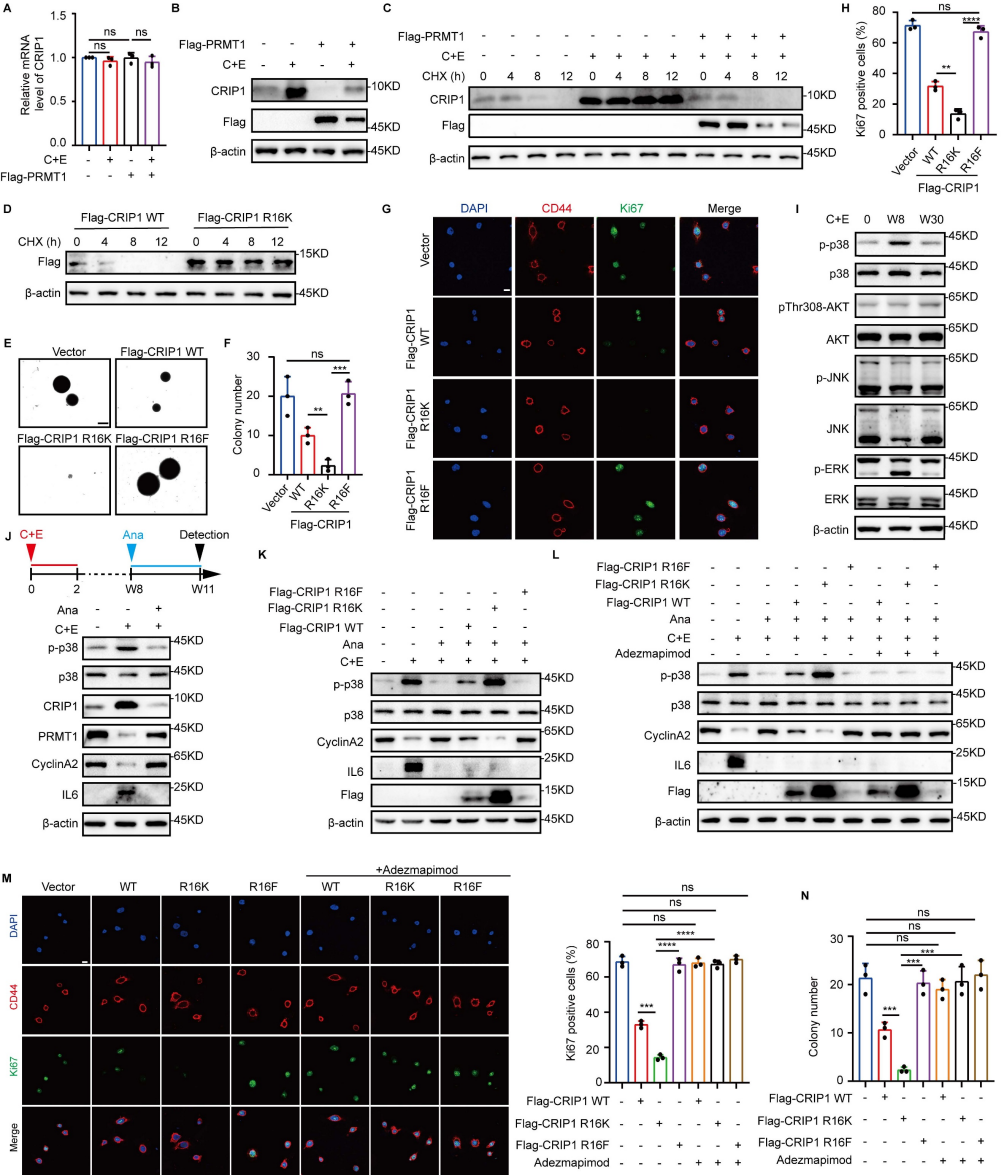
** PRMT1-mediated asymmetric dimethylation of CRIP1 promotes proliferation of stem-like cells.** (A) RT-qPCR analysis of CRIP1 on day W8 in NCI-H446 cells overexpressing Flag-PRMT1. (B) Western blot analysis of CRIP1 protein on day W8 in NCI-H446 cells overexpressing Flag-PRMT1. (C) Western blot analysis of CRIP1 half-life on day W8 in NCI-H446 cells overexpressing Flag-PRMT1, and treated with CHX. (D) Western blot analysis of Flag half-life in NCI-H446 cells overexpressing Flag-CRIP1 WT/R16K treated with CHX. (E and F) NCI-H446 cells overexpressing Flag-CRIP1 WT, R16K, or R16F at day W8 post-chemotherapy were cultured under low-adhesion conditions, seeded into soft agar, stained with crystal violet, photographed, and counted after 14 days (scale bar: 300 μm). (G and H) The enriched NCI-H446 stem-like cells overexpressing CRIP1 WT/R16K/R16F post-chemotherapy were analyzed to determine the proportion of Ki67-positive cells (scale: 10 μm). (I) Western blot analysis of proliferation-related proteins in NCI-H446 cells at indicated time points after chemotherapy. (J) Western blot analysis of p38, p-p38, CRIP1, PRMT1, CyclinA2, and IL6 on day W11 in NCI-H446 cells treated with C+E+Ana. (K) Western blot analysis of p-p38, p38, CyclinA2, IL6, and Flag on day W11 in NCI-H446 cells overexpressing Flag-CRIP1 WT/R16K/R16F with Ana from W8 to W11. (L) Western blot analysis of p-p38, p38, CyclinA2, IL6, and Flag on day W11 in NCI-H446 cells treated with Ana and Adezmapimod from W8-W11. (M) The enriched NCI-H446 stem-like cells after chemotherapy were seeded onto lysine-coated coverslips, treated with Adezmapimod for 72 hours, and the proportion of Ki67-positive cells was quantified. (N) NCI-H446 cells overexpressing Flag-CRIP1 WT, R16K, or R16F on day W8 post-chemotherapy were cultured under low-adhesion conditions, then seeded into soft agar treated with Adezmapimod. After 14 days, the cell clones were stained and counted (scale bar: 300 μm). Data are shown as the mean ± SD. **P < 0.01, ***P < 0.001 and ****P < 0.0001.

Our previous experiments have demonstrated that PRMT5-mediated methylation at the R26/68 residues of CRIP1 stabilizes CRIP1. Besides, upon knockdown of PRMT1, we observed a decrease in ASYM modification of CRIP1 concomitant with a significant increase in CRIP1 protein expression (Fig. 3H). To examine the role of PRMT1 in regulating CRIP1, we overexpressed PRMT1 and subsequently treated the cells with C+E. The results showed that CRIP1 protein expression was inhibited, with no corresponding change in mRNA levels (Fig. 5A and B). Furthermore, PRMT1 overexpression markedly shortened the half-life of CRIP1 (Fig. 5C). Strikingly, mutation at the R16 sites methylated by PRMT1 on CRIP1 led to an extension of the half-life of the protein (Fig. 5D). These results suggest PRMT1-mediated methylation of R16 residue reduces the protein stability of CRIP1.

CRIP1 inhibits breast cancer cell proliferation 21, which led us to hypothesize that PRMT1-mediated methylation of CRIP1 may affect the proliferation of stem-like cells during the late stage of recurrence. The proliferative capacity of stem-like cells after chemotherapy was assessed in cells overexpressing CRIP1 WT, CRIP1 R16K, and CRIP1 R16F. We found that SCLC stem-like cells overexpressing CRIP1 WT and CRIP1 R16F and treated with C+E exhibited faster proliferation rates than those overexpressing CRIP1 R16K (Fig. 5E-H). In addition, overexpression of CRIP1 R16F reversed the inhibitory effect of PRMT1 knockdown on the proliferation of SCLC at the late recurrence stage Fig. S5A). To elucidate the mechanism by which CRIP1 methylation at R16 site promoted stem-like cell proliferation, we examined the activation status of the p38, AKT, JNK, and ERK proliferation-related pathways at different time points, which showed that p38 phosphorylation was significantly inhibited in the late recurrent stage (Fig. 5I). Meanwhile, treatment with Ana on day W8 to block inflammation results in inhibition of p38 phosphorylation on day W11, along with an upregulation of PRMT1 and cyclinA2 (Fig. 5J, Fig. S5B), suggesting that the p38 pathway may play a role in the rapid proliferation of SCLC during the late recurrence stage.

Furthermore, we have further investigated the regulatory role of CRIP1 R16 methylation in p38 pathway activation. The result demonstrated that there was a direct interaction between the CRIP1 protein and p38 (Fig. S5C). It has been reported that MKK3 and MKK6 are key upstream kinases regulating the phosphorylation and activation of p38 (36. The result of co -immunoprecipitation revealed that CRIP1 interacted with p38 and its upstream kinase MKK3 (but not MKK6) Fig. S5D). Furthermore, CRIP1 WT and R16F suppressed the interaction between p38 and MKK3 compared to the R16K mutant (Fig. S5E). These findings suggest that methylation at the CRIP1 R16 site is likely involved in modulating p38 pathway activation. Notably, compared to WT and R16F, CRIP1 R16K overexpression inhibited the downregulation of p38 phosphorylation and the upregulation of CyclinA2 expression caused by the reduction of inflammation due to Ana addition (Fig. 5K, Fig. S5F-H). However, when further treated with the p38 inhibitor Adezmapimod on this basis, the inhibitory effect of R16K on cyclinA2 protein expression, as well as the subsequent inhibition of stem-like cell proliferation, was eliminated (Fig. 5L-N). These results demonstrate that PRMT1 promoted the rapid proliferation of stem-like cells by inhibiting the p38 pathway through asymmetric dimethylation of R16 of CRIP1.

### Inflammation activates E3 ubiquitin ligase PELI1 to mediate differential alterations of PRMT1 and PRMT5

**Figure 6:**
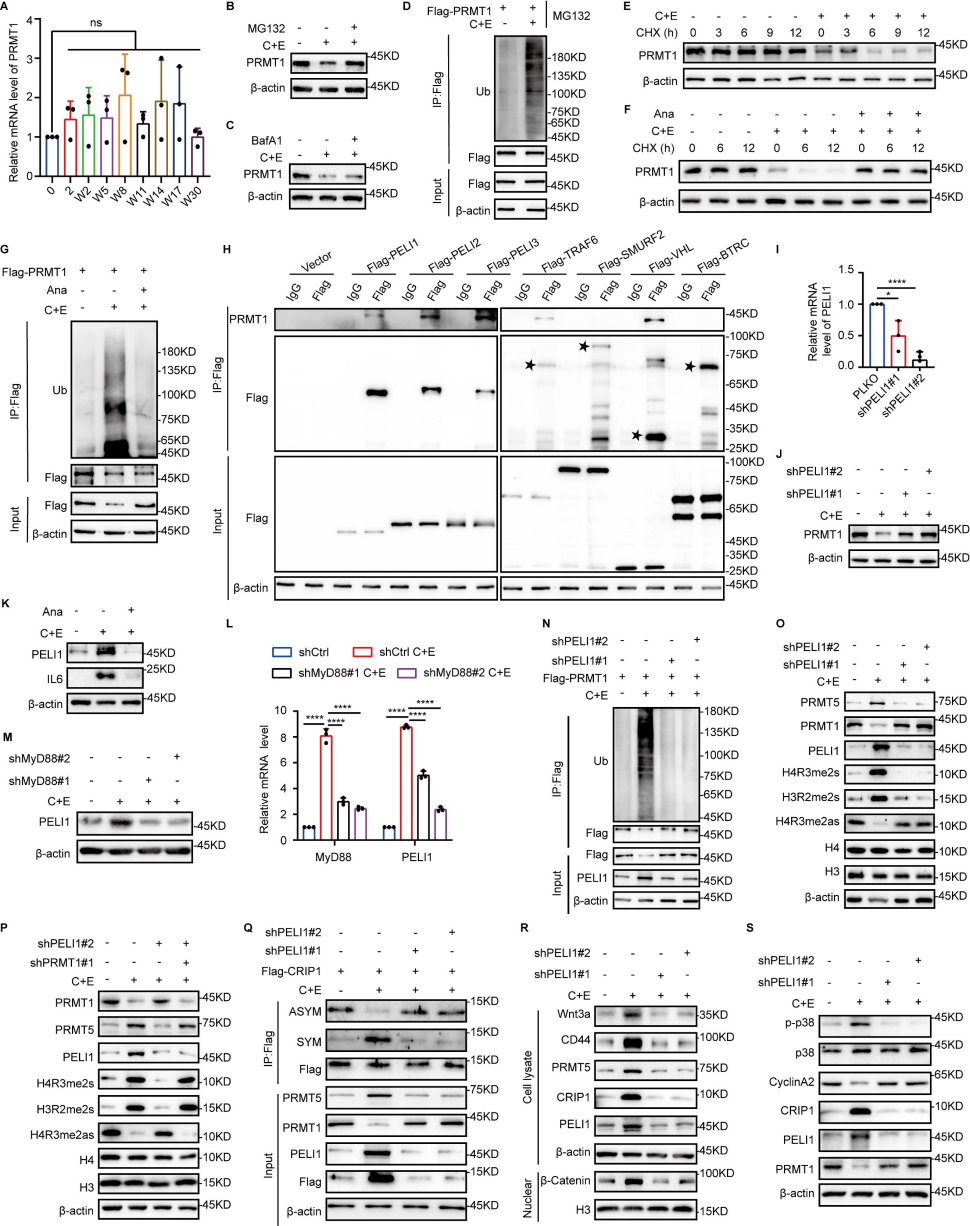
** Inflammation activates E3 ubiquitin ligase PELI1 to mediate differential alterations of PRMT1 and PRMT5.** (A) mRNA levels of PRMT1 in NCI-H446 cells at indicated time points after chemotherapy. (B and C) NCI-H446 cells on day W8 were treated with MG132 or BafA1 for 10 hours, and PRMT1 protein expression was detected by Western blot. (D) Ubiquitination of PRMT1 was detected in NCI-H446 cells overexpressing Flag-PRMT1 on day W8 after chemotherapy. (E) NCI-H446 cells on day W8 were treated with CHX, and PRMT1 was detected by Western blot. (F) NCI-H446 cells were co-treated with C+E+Ana, and subsequently analyzed for PRMT1 expression on day W8 after the addition of CHX. (G) Ubiquitination of PRMT1 was detected on day W8 in NCI-H446 cells co-treated with C+E+Ana. (H) E3 ubiquitin ligase binding to PRMT1 was detected in HEK-293T cells. (I and J) PELI1 interference efficiency was assessed by RT-qPCR. PRMT1 protein expression was detected by Western blot in PELI1-knockdown NCI-H446 cells on day W8. (K) PELI1 and IL6 protein levels were detected in NCI-H446 cells co-treated with C+E+Ana on day W8. (L and M) MyD88 was knocked down in NCI-H446 cells, and the changes in PELI1 mRNA and protein levels were detected on day W8 after chemotherapy. (N) Ubiquitination of PRMT1 was detected in PELI1-knockdown NCI-H446-Flag-PRMT1 cells on day W8 after chemotherapy. (O) Protein levels of PRMT5, PRMT1, PELI1, H4R3me2s, H3R2me2s, and H4R3me2as were detected in PELI1-knockdown NCI-H446 cells on day W8. (P) PRMT5 expression and activity were detected on day W8 in NCI-H446 cells with additional PRMT1 interference on the basis of PELI1 knockdown. (Q) NCI-H446-Flag-CRIP1 cells with PELI1 knockdown, and Western blot analysis of arginine methylation modifications of CRIP1 on day W8 after chemotherapy. (R) After PELI1 interference in NCI-H446 cells, Western blot analysis of Wnt3a, CD44, PRMT5, CRIP1, and PELI1 in total lysate, and nuclear β-Catenin on day W8 after chemotherapy. (S) After PELI1 knockdown in NCI-H446 cells, Western blot analysis of p38, p-p38, CyclinA2, CRIP1, PELI1, and PRMT1 in W8 cells. Data are shown as the mean ± SD. ns=not significant and ****P < 0.0001.

Next, we examined the effect of inflammation on the differential regulation of PRMT1 and PRMT5 function after chemotherapy. The results revealed that chemotherapy induced changes in PRMT1 protein expression while having no impact on its mRNA levels (Fig. 6A), indicating that PRMT1 is regulated at the post-translational level after chemotherapy. We treated W8 cells with the proteasome inhibitor MG132 and the autophagy inhibitor Bafilomycin A1 (BafA1) and found that MG132 restored PRMT1 expression (Fig. 6B and C). In addition, PRMT1 ubiquitination was significantly increased in W8 cells, and the half-life of PRMT1 was significantly shortened on day W8 after chemotherapy. (Fig. 6D and E). Treatment with Ana to inhibit post-chemotherapy inflammation significantly increased the half-life of PRMT1 and markedly suppressed its ubiquitination (Fig. 6F and G). These results indicate that post-chemotherapy inflammation reduced PRMT1 stability.

To further investigate the mechanism by which post-chemotherapy inflammation impacts PRMT1 stability, we selected several E3 ubiquitin ligases associated with inflammation and found that PELI1, PELI2, PELI3, TRAF6, and VHL bind to PRMT1 (Fig. 6H). We silenced each of these E3 ubiquitin ligases and found that knockdown of PELI1 significantly upregulated PRMT1 expression (Fig. 6I and J, Fig. S6A-D), and the expression of PELI1 was significantly suppressed following the inhibition of post-chemotherapy inflammation with Ana (Fig. 6K). During the research on the regulatory relationship between inflammation and PELI1, it was found that MyD88, serving as a crucial adaptor molecule in the inflammatory signaling pathway (37, was knocked down, which led to a significant inhibition of both PELI1 mRNA levels and protein expression (Fig. 6L and M). The results indicated that post-chemotherapy inflammation transcriptionally activated PELI1 through the MyD88 signaling pathway. Additionally, we found that PELI1 directly binds to PRMT1 Fig. S6E), and overexpression of PELI1 enhanced the ubiquitination level of PRMT1 (Fig. S6F). Knockdown of PELI1 significantly inhibited the ubiquitination-mediated degradation of PRMT1 in response to inflammation after chemotherapy (Fig. 6N). These findings further demonstrated that post-chemotherapy inflammation activated PELI1 to promote the ubiquitination and degradation of PRMT1.

Meanwhile, we found that knockdown of PELI1 increased the expression of PRMT1 while significantly inhibiting the expression of PRMT5 (Fig. 6O). However, knockdown of PRMT1 together with inhibition of PELI1 relieved the inhibitory effect of PELI1 interference on PRMT5 (Fig. 6P). The results suggest that the positive regulation of PRMT5 by PELI1 after chemotherapy may rely on PRMT1. To further clarify the regulatory relationships among PELI1, PRMT1, and PRMT5, the results of co-immunoprecipitation revealed that both PRMT5 and PELI1 interact with PRMT1 (Fig. S6G-I). Overexpression of PELI1 significantly inhibited the interaction between PRMT1 and PRMT5 (Fig. S6J). In cells with PRMT1 overexpression or PRMT5 knockdown, it was further confirmed that overexpression of PRMT1 following chemotherapy suppressed the protein level of PRMT5 (Fig. S6K and L). These findings reveal that the elevated PELI1 after chemotherapy not only promotes the ubiquitin-mediated degradation of PRMT1 but also relieves the inhibitory effect of PRMT1 on PRMT5, thereby achieving differential regulation of PRMT1 and PRMT5.

Furthermore, interference of PELI1 demonstrated that the regulatory effects of PELI1 on PRMT1 and PRMT5 further influenced the differential changes in arginine methylation modifications of CRIP1 (Fig. 6Q), thereby enabling PRMT5 and PRMT1 to play functional roles in promoting SCLC stemness characteristics and proliferation-promoting effects, respectively, at different stages of SCLC recurrence (Fig. 6R and S).

### Combination of PRMT5 inhibitor with cisplatin and etoposide delays the recurrence of SCLC

**Figure 7:**
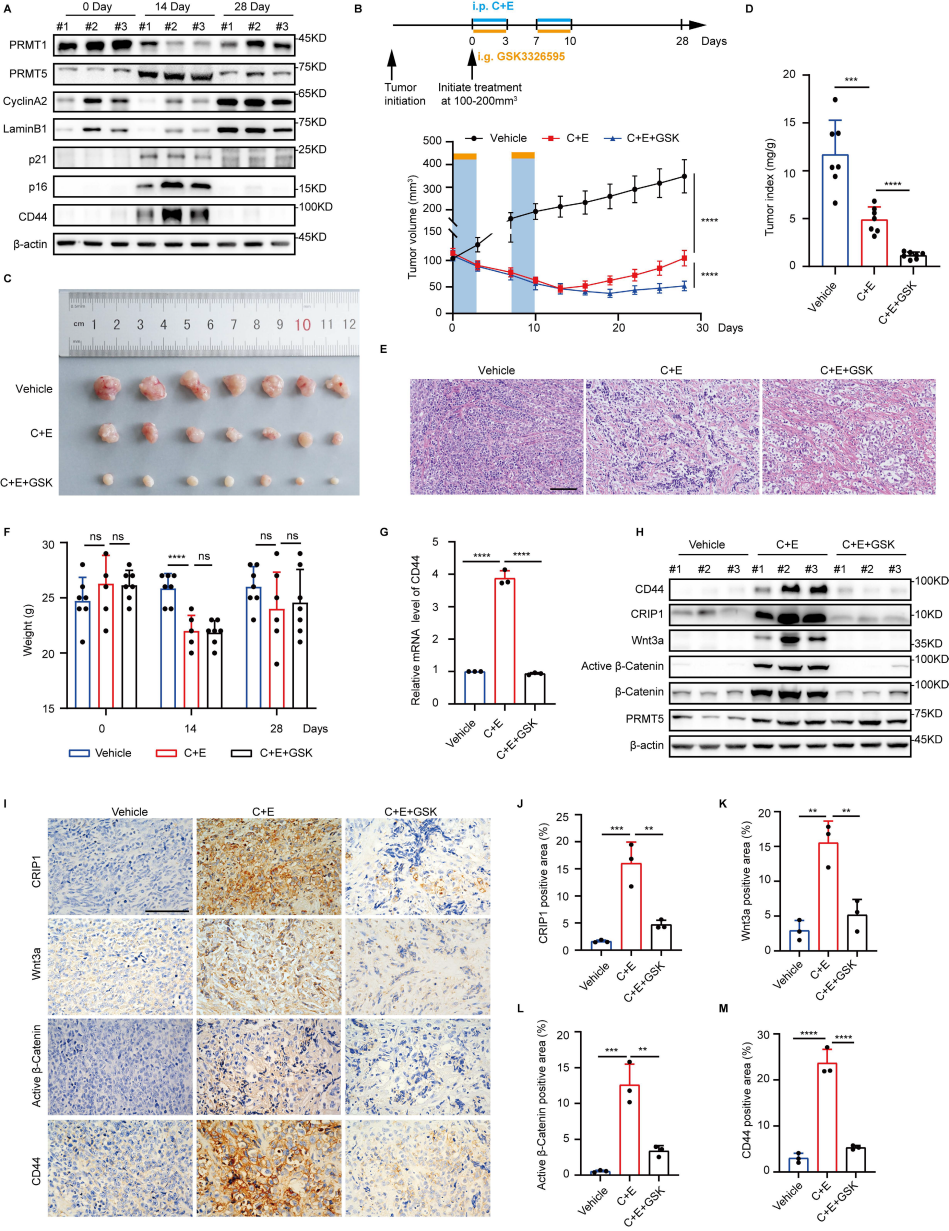
** Combination of PRMT5 inhibitor with cisplatin and etoposide delays the recurrence of SCLC.** (A) Tumors from the NCI-H446 xenograft model were collected pre-chemotherapy, on day 14, and day 28 post-treatment. Western blot detected protein expression. (B) Growth curve of NCI-H446 xenograft tumor volume. Treatment with C+E+GSK started when tumors reached 100-200 mm³. One therapy cycle spanned 7 days, with cisplatin administered on day 1, and etoposide along with GSK3326595 given on days 1-3. The therapy was continued for two cycles (n=7 mice). (C and D) Representative photograph of tumors and tumor index (tumor mg/weight g) at 28 days of treatment, with n=7 mice per group. (E) HE staining of mouse tumors on day 14 observed histopathological conditions (Scale bar: 100 μm). (F) Mouse body weight before treatment (0 Day), after treatment (14 Day), and at recurrence (28 Day), with n=7 mice per group. (G) On day 14 post-combination treatment, tumors were collected and RT-qPCR analysis of CD44 mRNA levels. (H) Western blot detected CD44, CRIP1, Wnt3a, β-Catenin, and Active β-Catenin protein levels in tumors collected on day 14 post-treatment. (I-M) Immunohistochemical analysis and statistical charts of positive proportions of CRIP1, Wnt3a, Active β-Catenin, and CD44 in tumor tissues among therapy groups on day 14 (Scale bar: 100 μm). Data are shown as the mean ± SD. ns=not significant, ***P < 0.001 and ****P < 0.0001.

Consistent with the *in vitro* findings, we also observed differential changes in the expression levels of PRMT1 and PRMT5 after chemotherapy in the NCI-H446 xenograft tumor mouse model, along with enhanced senescence and stemness phenotypes accompanying the chemotherapy (Fig. 7A). Differential modifications of CRIP1 by PRMT1 and PRMT5 contribute to SCLC recurrence after chemotherapy. Given that there are no specific *in vivo* inhibitors of PRMT1, we used the PRMT5-specific inhibitor GSK3326595 to suppress the stem-like phenotype of senescent cells following chemotherapy. On day 28 post-treatment, tumors in the C+E+GSK group were significantly smaller than those in the C+E group, and the tumor/body weight ratio was significantly reduced (Fig. 7B-D), indicating that the combination therapy with GSK slowed tumor recurrence.

Furthermore, following the completion of two rounds of therapy (on day 14), a marked reduction in the density of tumor tissues was observed, accompanied by extensive cell lysis and necrosis (Fig. 7E). Notably, there was no significant difference in the body weight of mice between the C+E+GSK group and the C+E group (Fig. 7F). Additionally, the combination therapy with GSK led to a decrease in the expression of stemness markers and the activity of the Wnt/β-catenin signaling pathway after C+E chemotherapy (Fig. 7G-M). Taken together, these results indicate that the combination of chemotherapy with GSK delays SCLC recurrence by inhibiting the stem-like phenotype of SCLC cells after chemotherapy.

## Discussion

**Figure 8:**
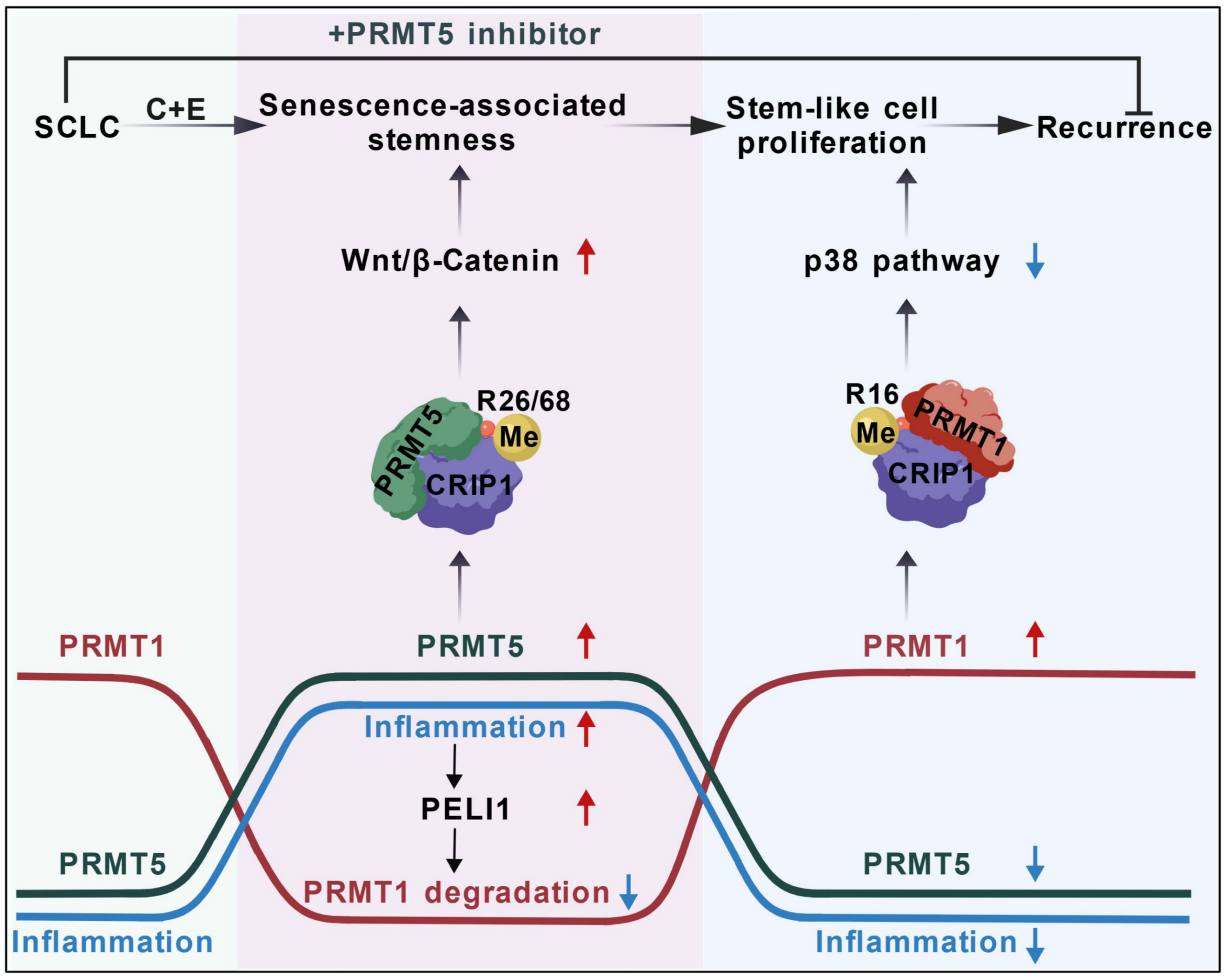
** Schematic diagram showing the differential arginine methylation of CRIP1 promotes the recurrence of SCLC after Chemotherapy.** The recurrence of SCLC after chemotherapy is accompanied by dynamic changes in inflammation. With an increase in inflammation levels, PRMT5-mediated methylation of CRIP1 at R26/68 promotes the acquisition of stem-like phenotypes in SCLC. When accompanied by a decrease in inflammation levels, PRMT1-mediated methylation of CRIP1 at R16 facilitates rapid proliferation of SCLC stem-like cells. Inflammation differentially regulates PRMT1 and PRMT5 through the activation of PELI1. The combination of PRMT5 inhibitors with chemotherapeutic drugs can delay the recurrence of SCLC.

The mechanisms underlying the high recurrence rate of SCLC following chemotherapy have remained largely unexplored. We found the differential changes between PRMT1 and PRMT5 in SCLC cells after cisplatin and etoposide treatment. PRMT5 increased at the early stage and then decreased at the later stage, while PRMT1 first decreased and then increased, which was regulated by an inflammation activated E3 ubiquitin ligase PELI1. Both PRMT5 and PRMT1 could modify the same substrate CRIP1. At the early stage, PRMT5-mediated CRIP1 R26/68 methylation activated the Wnt/β-catenin pathway to facilitate the acquisition of a stemness phenotype in senescent cells. At the later stage, PRMT1-mediated CRIP1 methylation at R16 residue suppressed the p38 pathway, further accelerating the proliferation of stem-like cells, thereby driving rapid recurrence of SCLC post-chemotherapy. Based on this, we used PRMT5 inhibitor combined with chemotherapy for SCLC, effectively delaying post-chemotherapy recurrence (Fig. 8.

PRMT1 and PRMT5 are aberrantly expressed in many tumors and have synergistic tumor-promoting effects 11, 12, 38. Here, we showed that PRMT5 and PRMT1 promoted the acquisition of a stemness phenotype in senescent cells and induced the rapid proliferation of stem-like cells after chemotherapy, thereby acting synergistically to promote the recurrence of SCLC. Tumor recurrence is often accompanied by epigenetic reprogramming of histones and non-histone proteins 39, 40. We showed that PRMT1 and PRMT5 regulated the process of SCLC recurrence after chemotherapy by catalyzing arginine methylation of CRIP1 R16 and R26/68. In addition, PRMT1 and PRMT5 methylate the same site, H4R3, on histone H4; PRMT1 acts as a transcriptional activator, whereas PRMT5 is a transcriptional repressor 41. These differential dynamic changes of PRMT1 and PRMT5 may regulate the transcriptional activation and repression of the same gene at different stages during SCLC recurrence through the differential modification of H4R3. Furthermore, PRMT5 methylates histone H3 at the R2 position and is involved in the transcriptional activation of cancer stemness-related genes 42, whereas PRMT1-mediated methylation of H4R3 activates the transcription of proliferation-related genes 43. This suggests that in addition to their differential regulation of the same gene, PRMT5 and PRMT1 play a role in the transcriptional activation of stemness-related genes and proliferation-related genes, respectively. Thus, the synergistic effect of PRMT1 and PRMT5 on SCLC recurrence after chemotherapy may be the result of their joint regulation at both the histone and non-histone levels.

Cellular senescence was initially considered an irreversible and effective approach to suppress tumor growth 44. However, numerous studies have found that TIS cells can evade cell cycle arrest by acquiring stem cell-like properties, thereby promoting tumor growth. For instance, in a doxorubicin-induced lymphoma senescence model, senescence-escaped lymphomas exhibited enhanced stem cell characteristics 7. Nevertheless, the underlying mechanisms by which senescent cells acquire stem-like features remain largely unknown. Our study revealed that the dynamic regulation of CRIP1 arginine methylation by PRMT5 and PRMT1 promoted senescence-associated stemness characteristics and the rapid proliferation of stem-like cells in SCLC following chemotherapy. Additionally, research has indicated that senescence-associated chromatin remodeling facilitated cancer stemness 7, and epigenetic genome reprogramming is recognized as a central event in the generation of induced pluripotent stem cells 45. Therefore, the findings of this study and existing relevant research collectively demonstrate that the mechanisms by which senescent cells evade suppression and acquire stem cell-like properties to promote tumor growth involve multiple aspects, including the dynamic regulation of arginine methylation, senescence-associated chromatin remodeling, and epigenetic reprogramming. These insights provide crucial clues and directions for a deeper understanding of the mechanisms underlying tumor initiation and progression.

The effect of CRIP1 on tumor progression is controversial. In ovarian cancer and acute myeloid leukemia, high expression of CRIP1 is closely associated with poor prognosis 19, 46. However, in osteosarcoma and breast cancer, high expression of CRIP1 is associated with antitumor effects 20, 21. We found that symmetric dimethylation of CRIP1 at R26/68 promotes the acquisition of stemness characteristics in senescent cells after chemotherapy, whereas asymmetric dimethylation at R16 of CRIP1 facilitates rapid proliferation of stem-like cells. The different arginine methylation modifications of CRIP1 enable it to play distinct functional roles in the recurrence process of SCLC, synergistically promoting SCLC recurrence after chemotherapy. The present results also indicate that ASYM and SYM modifications of CRIP1 have distinct effects on the stability of the protein. PRMT1 and PRMT5 differentially regulate the stability of CFLARL protein by modulating its interaction with the E3 ubiquitin ligase ITCH in human lung cancer 47. This finding indicates that the differential arginine methylation of CRIP1 by PRMT1 and PRMT5 may affect its binding to E3 ubiquitin ligases or deubiquitinating enzymes, thereby exerting differential effects on the stability of CRIP1. Further research is needed to clarify the effect of the differential modifications of CRIP1 by PRMT1 and PRMT5 on its stability.

The dual role of the inflammatory microenvironment in tumor progression has been reported. Inflammation can induce cancer cell death 48, 49, but inflammatory factors can also promote tumor growth and dissemination by enhancing the stem-like properties of cancer cells 8. We found that although increased inflammation following chemotherapy promoted the acquisition of a stemness phenotype in senescent cells, persistently high inflammation levels decreased the proliferation of stem-like cells. These dynamic changes in inflammation after chemotherapy contribute to the recurrence of SCLC. Furthermore, mechanistic studies revealed that post-chemotherapy inflammation promoted SCLC recurrence by mediating differential changes in the epigenetic modifying enzymes PRMT1 and PRMT5. Our results further explained the promoting effect of inflammation on tumorigenesis and progression from the perspective of epigenetic modifying enzymes. However, the source of this inflammation needs to be investigated further. In this study, we found that chemotherapy causes a large proportion of SCLC to enter a state of senescence. Senescent cells can secrete the Senescence-Associated Secretory Phenotype, which generates an inflammatory microenvironment 50. Our previous research also indicated that some senescent cells in SCLC induced by cisplatin and etoposide undergo pyroptosis, which is a type of inflammatory cell death 51. Therefore, cellular senescence and pyroptosis following chemotherapy may jointly contribute to the high levels of inflammation observed post-treatment.

In this study, the dynamic changes in inflammation and the arginine methyltransferases PRMT1 and PRMT5 that occur during the recurrence of SCLC were demonstrated at both the cellular level and in xenograft tumor models; however, our study lacks validation from clinical samples. Most SCLC patients already present with metastases at initial diagnosis 52, 53. Due to the high sensitivity of SCLC to chemotherapy, this treatment is prioritized over surgery, directly leading to difficulties in obtaining tissue samples and a scarcity of accessible public databases. Moreover, in our investigation of post-chemotherapy recurrence, collecting multi-stage clinical samples from treatment completion to recurrence is particularly challenging. The next step will involve using SCLC patient tumors to establish patient-derived xenograft (PDX) models to simulate post-chemotherapy recurrence and investigate dynamic changes in inflammatory levels and arginine methylation modifications after chemotherapy.

PRMT1 and PRMT5 are considered potential targets for inhibiting tumor development and progression in various types of cancer 54, 55. Despite the prior unavailability of specific inhibitors targeting PRMT1 and the recent development of GSK3368715 as a highly potent inhibitor, the Phase 1 clinical trial employing GSK3368715 for PRMT1-targeted therapy was prematurely terminated due to its suboptimal clinical efficacy and extensive treatment-associated adverse effects 56. However, the selective PRMT5 inhibitor, such as EPZ015938 (GSK3326595), has demonstrated favorable tumor-suppressing effects in both cell and animal models 57. Therefore, we used GSK3326595 in combination with C+E, and the combination treatment inhibited the stem-like phenotype of SCLC and delayed the recurrence of SCLC after chemotherapy. PRMT5 inhibitors are currently undergoing clinical trials for various cancers 57. These findings highlight the potential of combining chemotherapy with PRMT5 inhibitors as a treatment strategy for SCLC. Furthermore, we will conduct toxicological assessments to further evaluate the safety profile of GSK3326595 in combination with chemotherapy, aiming to facilitate the scientific application of the combination therapy in clinical practice.

## Supplementary Material

Supplementary figures and tables.

## Abbreviations

SCLC: Small cell lung cancer
TIS: Therapy-induced senescence
PRMTs: protein arginine methyltransferases
CRIP1: Cysteine-rich intestinal protein 1
ASYM: Arginine asymmetric demethylation
SYM: symmetric demethylation
C+E: Cisplatin plus etoposide
Ana: Anakinra
GSK: GSK3326595
HE: hematoxylin-eosin
IHC: immunohistochemistry
FBS: Fetal bovine serum
PBS: phosphate-buffered saline
