# Supplementary material for: PRMT1/PRMT5-Mediated Differential Arginine Methylation of CRIP1 Promotes the Recurrence of Small Cell Lung Cancer after Chemotherapy
Source: Int J Biol Sci. 2025 Sep 22;21(13):5956–74. doi: 10.7150/ijbs.115225 (PMC12509902; doi:10.7150/ijbs.115225)
Supplement: Supplementary file 1 — Supplementary figures and tables. [file ijbsv21p5956s1.pdf]

Supplementary Materials for

PRMT1/PRMT5-Mediated Differential Arginine Methylation of CRIP1 Promotes the Recurrence of Small Cell Lung Cancer after Chemotherapy

Yu Han *et al.*

*Corresponding author Email: zhangy288@nenu.edu.cn; luj809@nenu.edu.cn

This PDF file includes:

Figure S1 to S6
Table S1 to S3

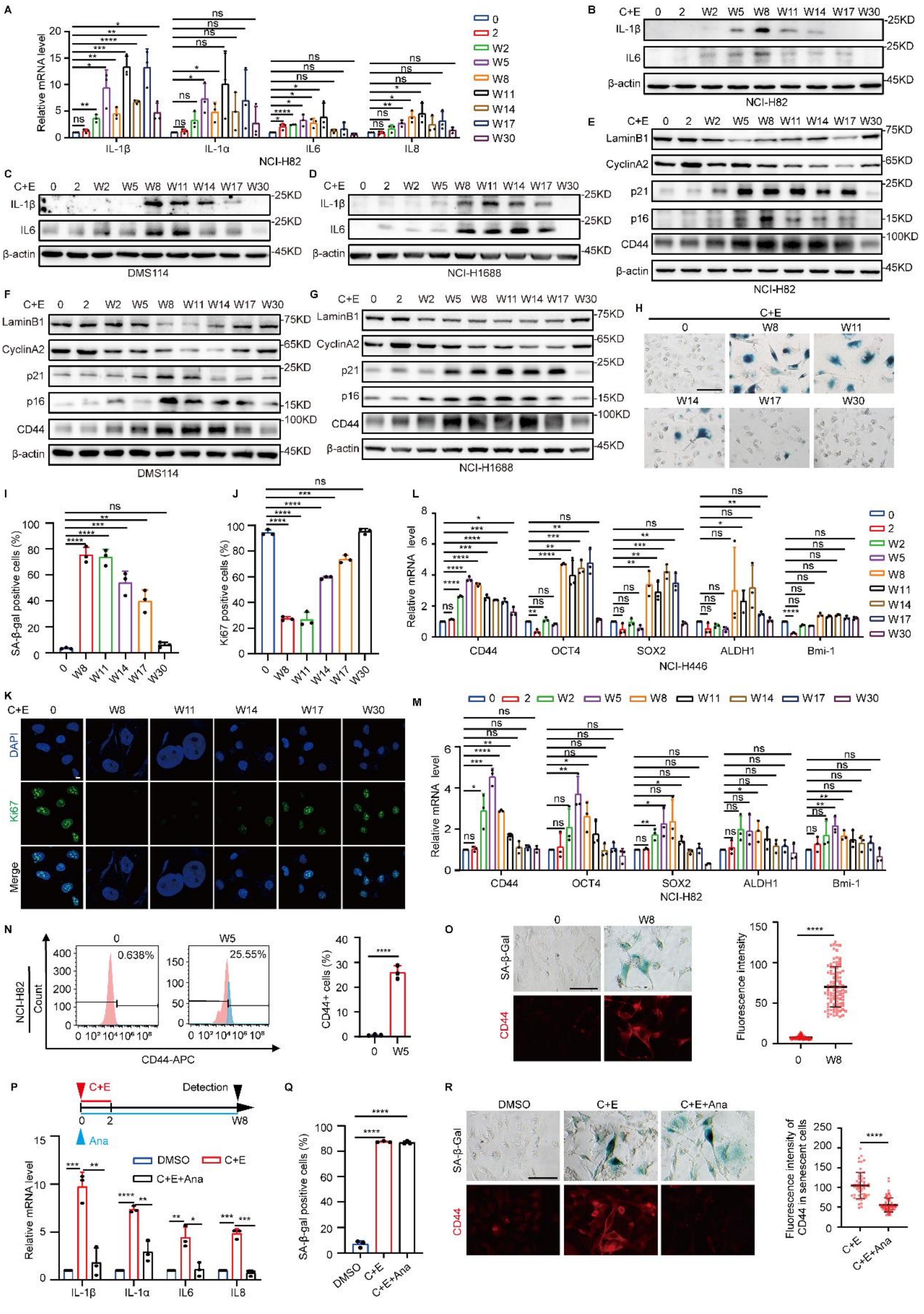

Figure S1. Recurrence of SCLC is accompanied by dynamic inflammatory changes after chemotherapy. (A) The mRNA levels of IL-1β, IL1α, IL6 and IL8 in NCI-H82 at different time points after chemotherapy. (B to D) Western blot analysis of IL-1β and IL6 protein expression in NCI-H82, DMS114 and NCI-H1688 at indicated time points after chemotherapy. (E to G) Western blot analysis of LaminB1, CyclinA2,

p21, p16 and CD44 protein expression in NCI-H82, DMS114 and NCI-H1688 at indicated time points after chemotherapy. (H and I) Quantification of SA-β-gal-positive cells in NCI-H446 at indicated time points after chemotherapy (scale bar: 100 μm). (J and K) Quantification of Ki67-positive cells in NCI-H446 at indicated time points after chemotherapy (scale bar: 10 μm). (L and M) The mRNA levels of CD44, OCT4, SOX2, ALDH1 and Bmi-1 in NCI-H446 and NCI-H82 at indicated time points after chemotherapy. (N) Flow cytometry analysis of CD44-positive cells in NCI-H82 before chemotherapy and on day W5 after chemotherapy. (O) Quantification of CD44 fluorescence intensity in NCI-H446 at indicated time points after chemotherapy. (scale bar: 100 μm). (P) Schematic of experimental design for C+E+ANA combination treatment. The mRNA levels of IL-1β, IL-1α, IL6 and IL8 were measured in NCI-H446 cells treated with C+E or C+E+Ana on day W8. (Q) The proportion of senescent cells was quantified by SA-β-gal staining (scale bar: 50 μm). (R) The fluorescence intensity of CD44 was measured in SA-β-gal-positive NCI-H446 cells treated with C+E+Ana on day W8. Data are shown as the mean ± SD. *$P < 0.05$, **$P < 0.01$, ***$P < 0.001$ and ****$P < 0.0001$.

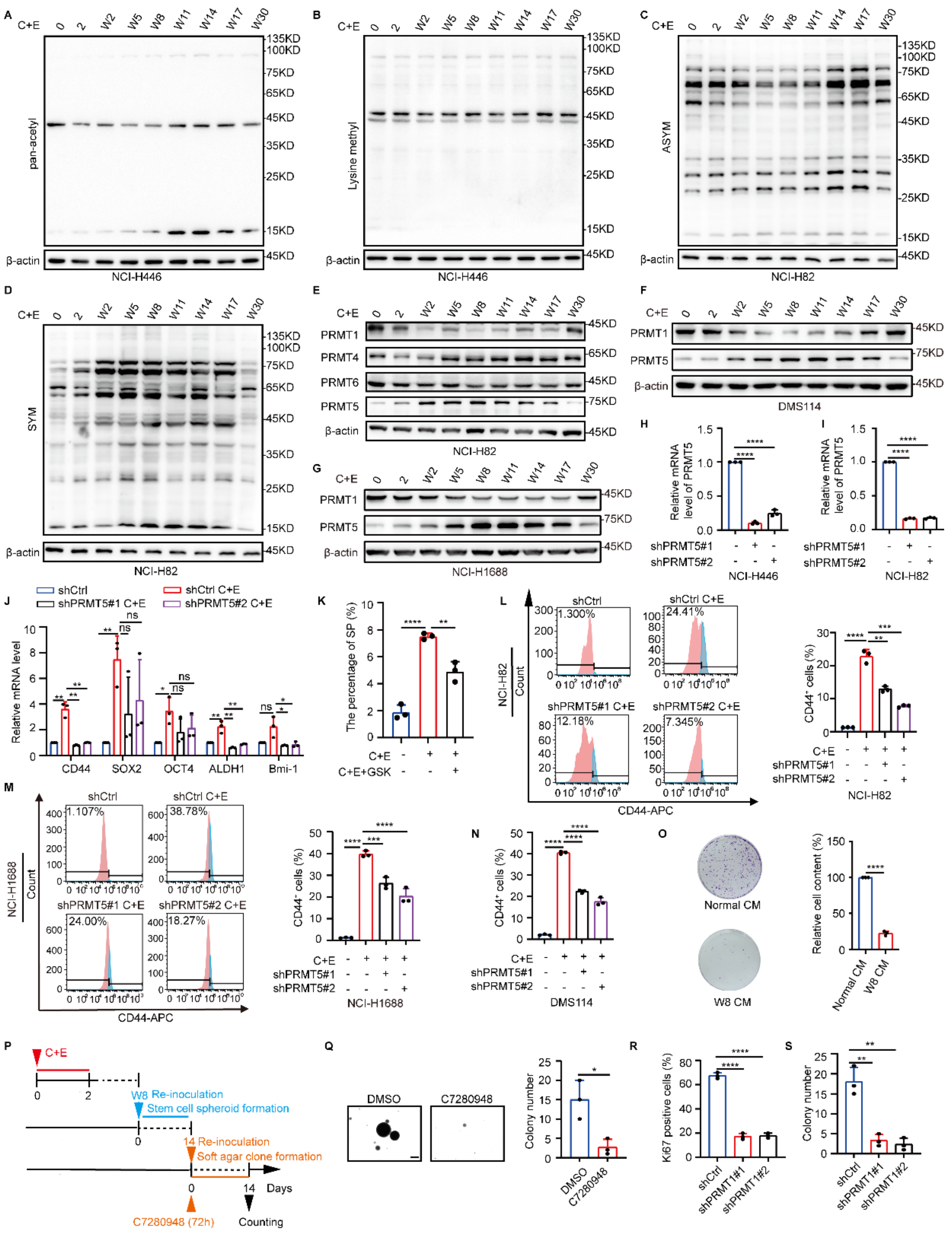

Figure S2. Inflammation levels regulate the differential alterations of PRMT1 and PRMT5. (A and B) Western blot analysis of pan-acetylation and lysine mono-/di-methylation in NCI-H446 cells. (C and D) Western blot analysis of ASYM and SYM of arginine in NCI-H82 at corresponding time points. (E to G) Western blot analysis of arginine methyltransferases expression in NCI-H82, DMS114 and NCI-H1688 at corresponding time points. (H to J) RT-qPCR analysis of PRMT5 interference efficiency in NCI-H446 and NCI-H82. After PRMT5 interference and treatment with C+E, RT-qPCR analysis of CD44, SOX2, OCT4, ALDH1 and Bmi-1 in NCI-H82 cells on day W5. (K) Flow cytometry analysis of the proportion of side population (SP) cells

on day W8 with C+E+GSK treatment in NCI-H446 cells. (L to N) Flow cytometry analysis of the proportion of CD44-positive cells in NCI-H82, DMS114 and NCI-H1688 cells with PRMT5 interference after chemotherapy. (O) NCI-H446 Cells were treated with W8 medium for W23-W30 cells, followed by crystal violet staining on day W30 for clone formation and quantification. (P and Q) On day W8 post-chemotherapy, stem cell sphere culture was performed in NCI-H446 cells to enrich the stem-like cells, which were then re-seeded into soft agar. C7280948 was added as a treatment for 72 hours during soft agar culture. The number of colonies was counted 14 days later (scale bar:300 μm). (R and S) After knocking down PRMT1 and enriching the stem-like cells of NCI-H446 after chemotherapy, the stem-like cells were reseeded onto lysine-coated coverslips, and the proportion of Ki67-positive cells was quantified after attachment. The stem-like cells were also inoculated into soft agar, and the number of colonies formed was counted 14 days later. Data are shown as the mean ± SD. *$P < 0.05$, **$P < 0.01$, ***$P < 0.001$ and ****$P < 0.0001$.

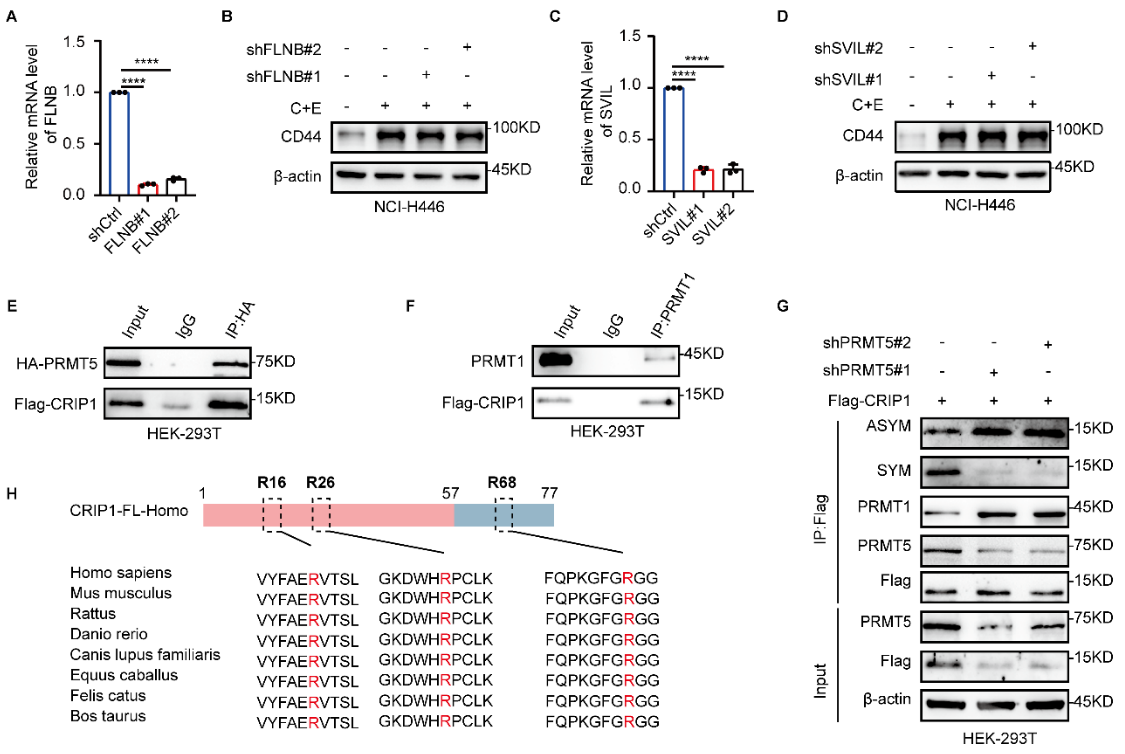

Figure S3. Inflammation levels mediate the competition between PRMT1 and PRMT5 for CRIP1 methylation. (A to D) FLNB or SVIL was knocked down in NCI-H446 cells, and RT-qPCR analysis of interference efficiency. Western blot analysis of CD44 protein expression in W8 cells after chemotherapy. (E) HEK-293T cells transfected with Flag-CRIP1 and HA-PRMT5, and co-immunoprecipitation and immunoblot analysis of the interaction between PRMT5 and CRIP1. (F) HEK-293T cells transfected with Flag-CRIP1, and co-immunoprecipitation and immunoblot analysis of the interaction between PRMT1 and CRIP1. (G) Flag-CRIP1-transfected HEK-293T cells with PRMT5 knocked down, and co-immunoprecipitation analysis of ASYM and SYM modifications on CRIP1, and its binding to PRMT1 and PRMT5. (H) Conservation of R16, R26, and R68 arginine sites in CRIP1 across species was analyzed. Data are shown as the mean ± SD. ****$P < 0.0001$.

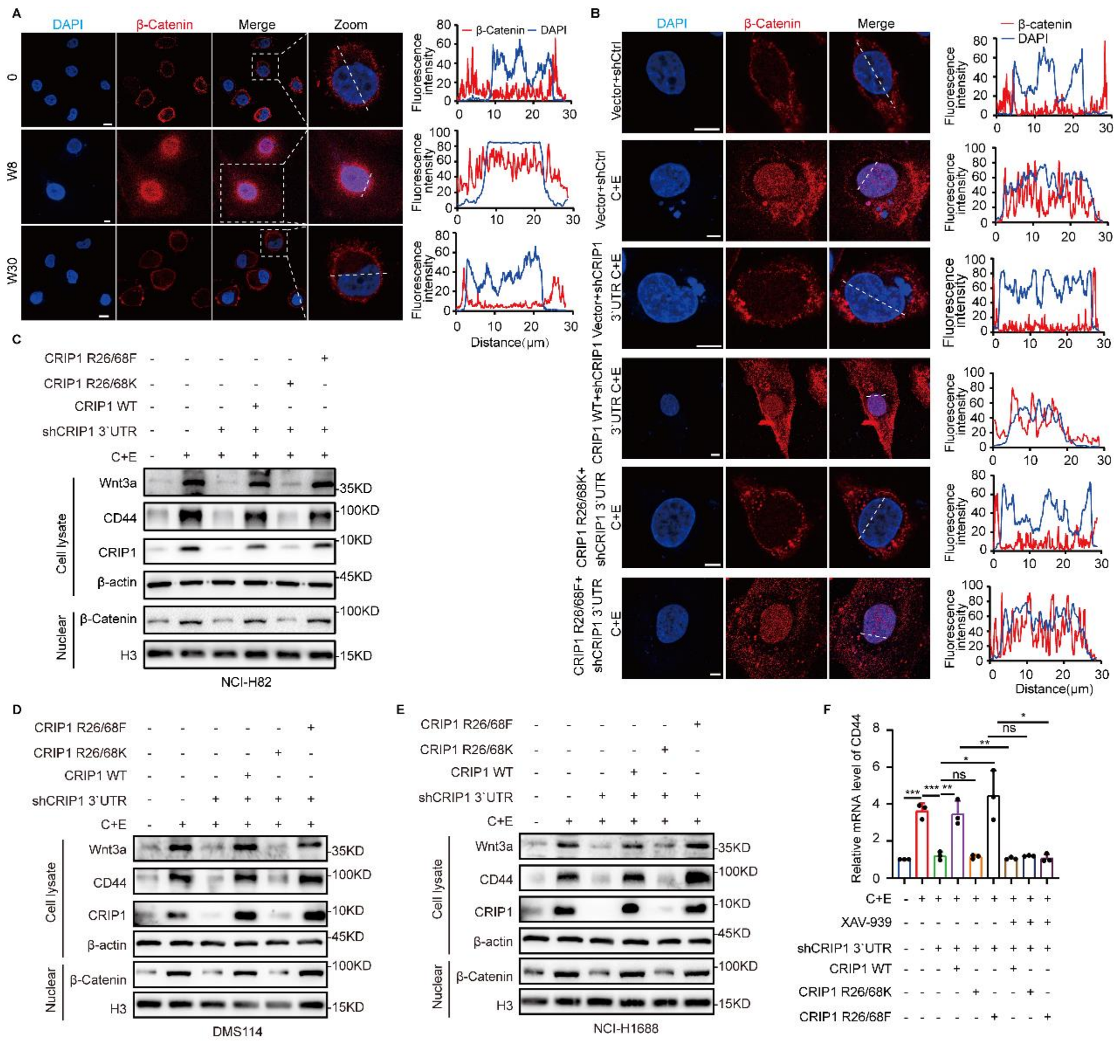

Figure S4. PRMT5-mediated symmetric dimethylation of CRIP1 promotes the acquisition of stemness phenotype. (A) β-Catenin fluorescent labeling was conducted in NCI-H446 cells before chemotherapy, as well as on day W8 and W30 to observe its localization (scale bar: 10 μm). (B) In NCI-H446 cells with CRIP1 knocked down, CRIP1 WT, R26/68K, and R26/68F were overexpressed. Immunofluorescence detected β-Catenin localization in W8 cells (scale bar: 10 μm). (C to E) In NCI-H82, DMS114 and NCI-H1688 cells with CRIP1 knocked down, CRIP1 WT, R26/68K, R26/68F were overexpressed. Western blot analysis of Wnt3a, CD44, and CRIP1 in total lysate, as well as β-Catenin in the nuclear extracts of cells on day W8. (F) RT-qPCR analysis of CD44 on day W8 in NCI-H446 cells treated with C+E+XAV-939. Data are shown as the mean ± SD. *$P < 0.05$, **$P < 0.01$ and ***$P < 0.001$.

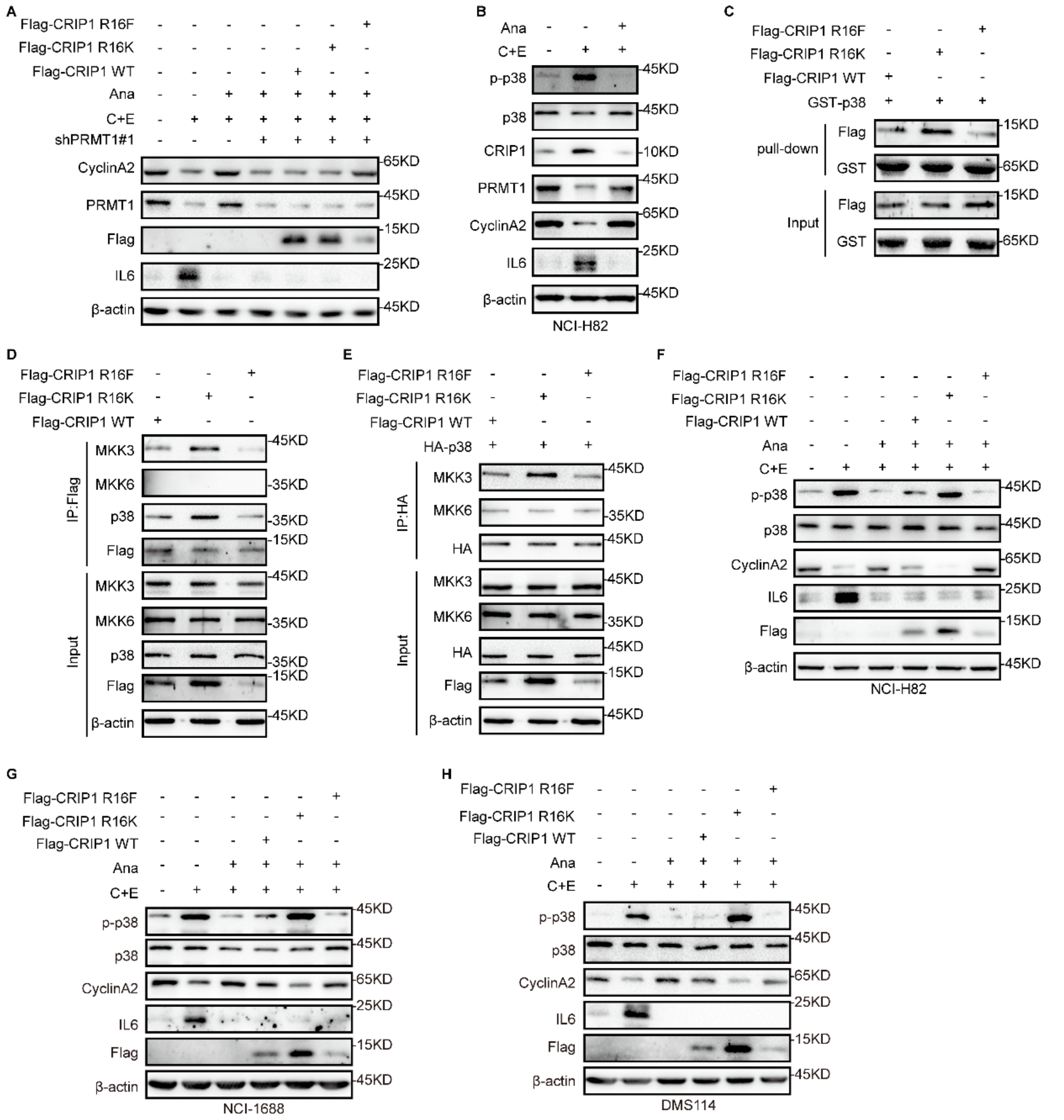

Figure S5. PRMT1-mediated asymmetric dimethylation of CRIP1 promotes proliferation of stem-like cells. (A) Western blot analysis of CyclinA2 on day W11 in NCI-H446 cells overexpressing Flag-CRIP1 WT, R16K, R16F after PRMT1 knockdown, treated with Ana from W8-W11. (B) On W8 post-chemotherapy in NCI-H82 cells, Ana was added, and Western blot analysis of p38, p-p38, CRIP1, PRMT1, CyclinA2, and IL6 in W11 cells. (C) GST-pulldown assay for binding of p38 to CRIP1 WT and CRIP1 mutants. (D) Co-immunoprecipitation analysis of the interactions between CRIP1 (WT and mutants) and p38/MKK3/MKK6 in NCI-H446 cells. (E) Co-immunoprecipitation analysis of p38 interactions with MKK3/MKK6 in NCI-H446 cells overexpressing Flag-CRIP1 (WT, R16K, R16F) and HA-p38. (F to H) In NCI-H82, DMS114 and NCI-H1688 cells overexpressing Flag-CRIP1 WT, R16K, R16F, post-chemotherapy and Ana addition on day W8, Western blot analysis of p-p38, p38, CyclinA2, IL6, and Flag in W11 cells.

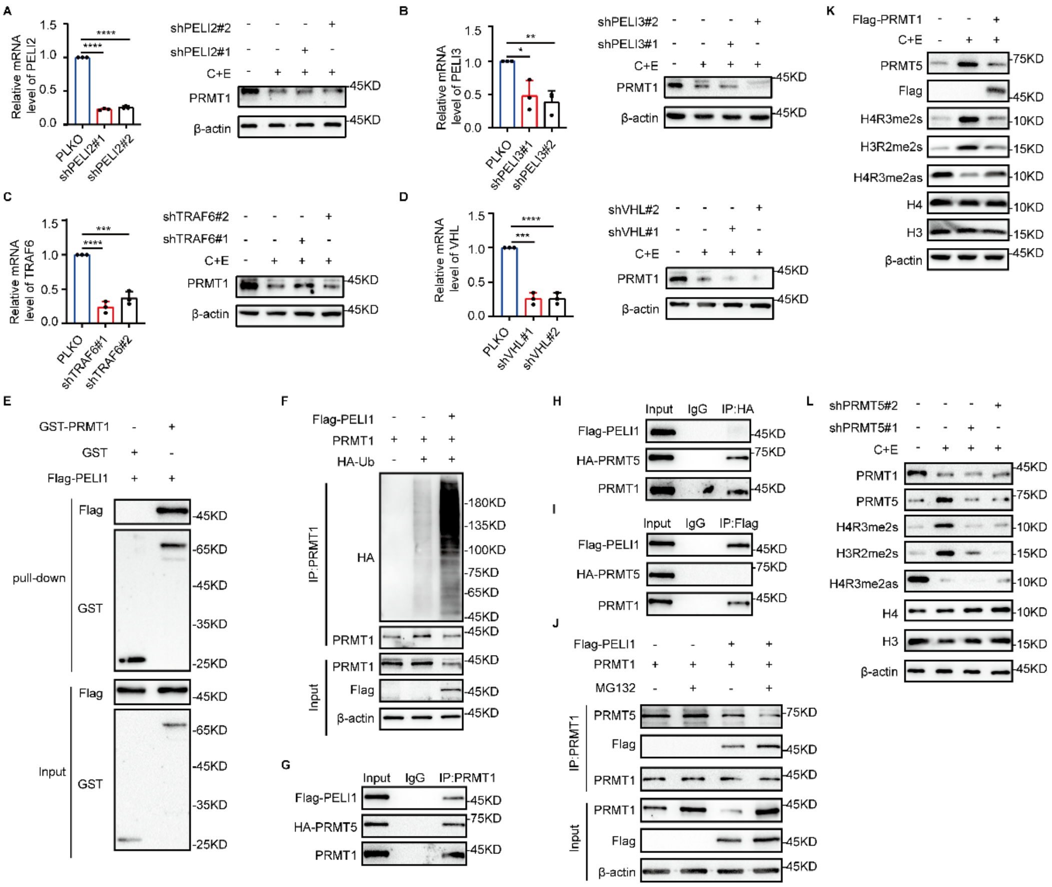

Figure S6. Inflammation activates E3 ubiquitin ligase PELI1 to mediate differential alterations of PRMT1 and PRMT5. (A to D) E3 ubiquitin ligase was knocked down in NCI-H446 cells, and interference efficiency was assessed by RT-qPCR. PRMT1 protein expression was then detected by Western blot in W8 cells post-interference. (E) GST pulldown assay was performed to detect PELI1-PRMT1 interaction. (F) Ubiquitination of PRMT1 was detected in HEK-293T cells overexpressing Flag-PELI1 and PRMT1. (G to I) Overexpression of Flag-PELI1 and HA-PRMT5 was conducted in HEK-293T cells, and co-immunoprecipitation assays were performed to examine the interactions among PRMT1, PRMT5, and PELI1. (J) Overexpression of Flag-PELI1 was performed in NCI-H446 cells, and binding of PRMT1 to either PRMT5 or PELI1 was detected after treatment with MG132. (K and L) PRMT1 was overexpressed or PRMT5 was knocked down in NCI-H446 cells, and changes in protein expression and activity of PRMT1 and PRMT5 were detected by Western blot on day W8. Data are shown as the mean ± SD. *$P < 0.05$, **$P < 0.01$, ***$P < 0.001$ and ****$P < 0.0001$.

Table S1. Antibody resources.

Antibodies	Source	Identifier
CD44	Abcam	ab254530
CD44	Abclonal	A19020
Wnt3a	Sangon Biotech	D222111
β-Catenin	BD Biosciences	610154
β-actin	Abclonal	AC026
Flag	Abmart	M20008
HA	Sigma-Aldrich	H9658
H4	Abcam	ab223875
Active β-Catenin	Abclonal	A22180
PRMT1	CST	2449
AKT	CST	9272
PRMT6	CST	14641
H4R3me2s	Abcam	ab5823
Ubquitin	CST	3933
H3	Abclonal	A17562
H3R2me2s	Abcam	ab194684
LaminB1	Abcam	ab16048
CyclinA2	CST	67955
Ki67	GeneTex	GTX16667
IL6	CST	12153
p21	Proteintech	10355-1-AP
p16	BD Biosciences	51-1325GR
IL1β	Abcam	ab216995
CRIP1	Proteintech	15349-1-AP
ASYM	Millipore	07-414
SYM	Millipore	07-413
PELI1	Proteintech	12053-1-AP
p-p38	CST	9211
p38	CST	9212
p-ERK	CST	4370
ERK	CST	4695
JNK	CST	9252
p-JNK	CST	4671
p-AKT	CST	13038
H4R3me2as	Abcam	ab194683
MKK3	MCE	HY-P83469
MKK6	Abclonal	A2575

Table S2. The shRNA primer sequences.

Plasmid	Primer sequences
shCtrl sense	CCGGAATGCCTACGTTAAGCTATACCTCGAGGTATAGCTTAACGTAGGCATTTTTTTG
shCtrl antisense	AATTCAAAAAAATGCCTACGTTAAGCTATACCTCGAGGTATAGCTTAACGTAGGCATT
shPRMT5#1 sense	CCGGCCCATCCTCTTCCCTATTAAGCTCGAGCTTAATAGGGAAGAGGATGGGTTTTTG
shPRMT5#1 antisense	AATTCAAAAACCCATCCTCTTCCCTATTAAGCTCGAGCTTAATAGGGAAGAGGATGGG
shPRMT5#2 sense	CCGGGCCCAGTTTGAGATGCCTTATCTCGAGATAAGGCATCTCAAACTGGGCTTTTTG
shPRMT5#2 antisense	AATTCAAAAAGCCCAGTTTGAGATGCCTTATCTCGAGATAAGGCATCTCAAACTGGGC
shPELI1#1 sense	CCGGCCATGTACATGGCTATCATAACTCGAGTTATGATAGCCATGTACATGGTTTTTG
shPELI1#1 antisense	AATTCAAAAACCATGTACATGGCTATCATAACTCGAGTTATGATAGCCATGTACATGG
shPELI1#2 sense	CCGGGCCAAATGGAAGACATCAGATCTCGAGATCTGATGTCTTCCATTTGGCTTTTTG
shPELI1#2 antisense	AATTCAAAAAGCCAAATGGAAGACATCAGATCTCGAGATCTGATGTCTTCCATTTGGC
shPELI2#1 sense	CCGGGCAAAGGTCAACACAGTATATCTCGAGATATACTGTGTTGACCTTTGCTTTTTG
shPELI2#1 antisense	AATTCAAAAAGCAAAGGTCAACACAGTATATCTCGAGATATACTGTGTTGACCTTTGC
shPELI2#2 sense	CCGGGAACCTTACACAGCACGGATACTCGAGTATCCGTGCTGTGTAAGGTTCTTTTTG
shPELI2#2 antisense	AATTCAAAAAGAACCTTACACAGCACGGATACTCGAGTATCCGTGCTGTGTAAGGTTC
shPELI3#1 sense	CCGGCGACACAGACATGTTCCAGATCTCGAGATCTGGAACATGTCTGTGTCGTTTTTG
shPELI3#1 antisense	AATTCAAAAACGACACAGACATGTTCCAGATCTCGAGATCTGGAACATGTCTGTGTCG
shPELI3#2 sense	CCGGCGATGCCTCTAGCAACATCTTCTCGAGAAGATGTTGCTAGAGGCATCGTTTTTG
shPELI3#2 antisense	AATTCAAAAACGATGCCTCTAGCAACATCTTCTCGAGAAGATGTTGCTAGAGGCATCG
shTRAF6#1 sense	CCGGCGGAATTTCCAGGAAACTATTCTCGAGAATAGTTTCCTGGAAATTCCGTTTTTG

Continuation of the Table S2

shTRAF6#1 antisense	AATTCAAAAACGGAATTTCCAGGAAACTATTCTCGAG AATAGTTTCCTGGAAATTCCG
shTRAF6#2 sense	CCGGCGAAGAGATAATGGATGCCAACTCGAGTTGGCA TCCATTATCTCTTCGTTTTTG
shTRAF6#2 antisense	AATTCAAAAACGAAGAGATAATGGATGCCAACTCGAG TTGGCATCCATTATCTCTTCG
shVHL#1 sense	CCGGTATCACACTGCCAGTGTATACCTCGAGGTATACA CTGGCAGTGTGATATTTTTG
shVHL#1 antisense	AATTCAAAAATATCACACTGCCAGTGTATACCTCGAGG TATACACTGGCAGTGTGATA
shVHL#2 sense	CCGGCAATGTTGACGGACAGCCTATCTCGAGATAGGCT GTCCGTCAACATTGTTTTTG
shVHL#2 antisense	AATTCAAAAACAATGTTGACGGACAGCCTATCTCGAG ATAGGCTGTCCGTCAACATTG
shCRIP1-3’UTR sense	CCGGGCTCTCAGTAAACCTGAACACCTCGAGGTGTTCA GGTTTACTGAGAGCTTTTTG
shCRIP1-3’UTR antisense	AATTCAAAAAGCTCTCAGTAAACCTGAACACCTCGAG GTGTTCAGGTTTACTGAGAGC
shCRIP1#1 sense	CCGGCAGCCATGTTTGGGCCTAAAGCTCGAGCTTTAGG CCCAAACATGGCTGTTTTTG
shCRIP1#1 antisense	AATTCAAAAACAGCCATGTTTGGGCCTAAAGCTCGAG CTTTAGGCCCAAACATGGCTG
shCRIP1#2 sense	CCGGAGCACGAAGGCAAACCCTACTCTCGAGAGTAGG GTTTGCCTTCGTGCTTTTTG
shCRIP1#2 antisense	AATTCAAAAAAGCACGAAGGCAAACCCTACTCTCGAG AGTAGGGTTTGCCTTCGTGC
shSVIL#1 sense	CCGGCCGAGTATTTATCCCGCTATACTCGAGTATAGCG GGATAAATACTCGGTTTTTG
shSVIL#1 antisense	AATTCAAAAACCGAGTATTTATCCCGCTATACTCGAGT ATAGCGGGATAAATACTCGG
shSVIL#2 sense	CCGGGCTACTTATATCCAAACCATTCTCGAGAATGGTT TGGATATAAGTAGCTTTTTG
shSVIL#2 antisense	AATTCAAAAAGCTACTTATATCCAAACCATTCTCGAGA ATGGTTTGGATATAAGTAGC
shFLNB#1 sense	CCGGCCAGAAATCAACAGCAGTGATCTCGAGATCACT GCTGTTGATTTCTGGTTTTTG
shFLNB#1 antisense	AATTCAAAAACCAGAAATCAACAGCAGTGATCTCGAG ATCACTGCTGTTGATTTCTGG
shFLNB#2 sense	CCGGCCTGTGGATAATGCACGAGAACTCGAGTTCTCGT GCATTATCCACAGGTTTTTG

Continuation of the Table S2

shFLNB#2 antisense	AATTCAAAAACCTGTGGATAATGCACGAGAACTCGAG TTCTCGTGCATTATCCACAGG
shMyD88#1 sense	CCGGGCAGAGCAAGGAATGTGACTTCTCGAGAAGTCA CATTCCTTGCTCTGCTTTTTG
shMyD88#1 antisense	AATTCAAAAAGCAGAGCAAGGAATGTGACTTCTCGAG AAGTCACATTCCTTGCTCTGC
shMyD88#2 sense	CCGGACAGACAAACTATCGACTGAACTCGAGTTCAGT CGATAGTTTGTCTGTTTTTG
shMyD88#2 antisense	AATTCAAAAAACAGACAAACTATCGACTGAACTCGAG TTCAGTCGATAGTTTGTCTGT

142 **Table S3. Gene-specific primers used in the RT-qPCR.**

Gene	Accession number	Primer sequences
β-actin	NM_001101.5	Forward: GAGCACAGAGCCTCGCCTTT
		Reverse: ATCCTTCTGACCCATGCCCA
IL6	NM_001318095.2	Forward: CCCCTGACCCAACCACAAAT
		Reverse: ATTTGCCGAAGAGCCCTCAG
IL8	NM_000584.4	Forward: GAGTGGACCACACTGCGCCA
		Reverse: TCCACAACCCTCTGCACCCAGT
IL1α	NM_000575.5	Forward: AACCAGTGCTGCTGAAGGA
		Reverse: TTCTTAGTGCCGTGAGTTTCC
IL1β	NM_000576.3	Forward: ATGATGGCTTATTACAGTGGCAA
		Reverse: GTCGGAGATTCGTAGCTGGA
PRMT1	NM_001207042.3	Forward: CCAGGCGGAAAGCAGTGAG
		Reverse: GGAGTTGCGGTAAGTGAGGGT
PRMT5	NM_001282953.2	Forward: CACCTTCAGCCATCCCAACAGAG
		Reverse: CCATGAGAACATCCCAGGAGAGTG
SVIL	NM_001323599.2	Forward: TTTCCAGCCTGTCCAACTTCA
		Reverse: CGTCACCTACTGCCATAACCC
CRIP1	NM_001311.5	Forward: CCTGCCTGAAGTGCGAGAAAT
		Reverse: CCTTTAGGCCCAAACATGGC
FLNB	NM_001164317.2	Forward: ACTGTCATGGCCACAGATGG
		Reverse: AAATCCCAGGCCGTTCATGT
PELI1	XM_011532994.4	Forward: CGGCTCAGCAGAGAGGAAAA
		Reverse: TCACGGTAGGAGTGTGGGAA
PELI2	NM_021255.3	Forward: CGCGCGCGGATTTGACTCTT
		Reverse: CTGGGTGAAGCCCCCTCGTG
PELI3	NM_145065.3	Forward: CTGGAAGGAAACCCTGAAGT
		Reverse: AGCGGCGTGGAGATGTG
TRAF6	NM_004620.4	Forward: GTTGCTGAAATCGAAGCACA
		Reverse: CGGGTTTGCCAGTGTAGAAT
VHL	NM_000551.4	Forward: GGAGCCTAGTCAAGCCTGAGA
		Reverse: CATCCGTTGATGTGCAATGCG
CD44	XM_054370571.1	Forward: AAGGTGGAGCAAACACAACC
		Reverse: AGCTTTTTCTTCTGCCCACA
SOX2	NM_003106.4	Forward: GCACATGAACGGCTGGAGCAACG
		Reverse: TGCTGCGAGTAGGACATGCTGTAGG
Bmi-1	NM_001428311.1	Forward: GACCACTACTGAATATAAGG
		Reverse: CATTTGTCAGTCCATCTCTC
OCT4	NM_203289.6	Forward: GAAGGATGTGGTCCGAGTGT
		Reverse: GTGAAGTGAGGGCTCCCATA

Continuation of the Table S3

ALDH	NM_000689.5	Forward: GTTAGCTGATGCCGACTTGG
		Reverse: CCCACTCTCAATGAGGTCAAG
MyD88	NM_001172567.2	Forward: CAGCGACATCCAGTTTGTGC
		Reverse: GGCCTTCTAGCCAACCTCTT
